# Combating viral contaminants in CHO cells by engineering innate immunity

**DOI:** 10.1038/s41598-019-45126-x

**Published:** 2019-06-20

**Authors:** Austin W. T. Chiang, Shangzhong Li, Benjamin P. Kellman, Gouri Chattopadhyay, Yaqin Zhang, Chih-Chung Kuo, Jahir M. Gutierrez, Faezeh Ghazi, Hana Schmeisser, Patrice Ménard, Sara Petersen Bjørn, Bjørn G. Voldborg, Amy S. Rosenberg, Montserrat Puig, Nathan E. Lewis

**Affiliations:** 1 Department of Pediatrics, University of California, San Diego, La Jolla, CA 92093 USA; 2 The Novo Nordisk Foundation Center for Biosustainability at the University of California, San Diego, La Jolla, CA 92093 USA; 3 Department of Bioengineering, University of California, San Diego, La Jolla, CA 92093 USA; 4 Bioinformatics and Systems Biology Graduate Program, University of California, San Diego, La Jolla, CA 92093 USA; 5 Center for Drug Evaluation and Research, U.S. Food and Drug Administration, Silver Spring, MD 20993 USA; 6 Viral Pathogenesis Section, Laboratory of Immunoregulation, National Institute of Allergy and Infectious Disease, National Institutes of Health, Bethesda, MD 20892 USA; 7 The Novo Nordisk Foundation Center for Biosustainability, Technical University of Denmark, Hørsholm, Denmark

**Keywords:** Virus-host interactions, Next-generation sequencing, Cellular signalling networks

## Abstract

Viral contamination in biopharmaceutical manufacturing can lead to shortages in the supply of critical therapeutics. To facilitate the protection of bioprocesses, we explored the basis for the susceptibility of CHO cells to RNA virus infection. Upon infection with certain ssRNA and dsRNA viruses, CHO cells fail to generate a significant interferon (IFN) response. Nonetheless, the downstream machinery for generating IFN responses and its antiviral activity is intact in these cells: treatment of cells with exogenously-added type I IFN or poly I:C prior to infection limited the cytopathic effect from Vesicular stomatitis virus (VSV), Encephalomyocarditis virus (EMCV), and Reovirus-3 virus (Reo-3) in a STAT1-dependent manner. To harness the intrinsic antiviral mechanism, we used RNA-Seq to identify two upstream repressors of STAT1: Gfi1 and Trim24. By knocking out these genes, the engineered CHO cells exhibited activation of cellular immune responses and increased resistance to the RNA viruses tested. Thus, omics-guided engineering of mammalian cell culture can be deployed to increase safety in biotherapeutic protein production among many other biomedical applications.

## Introduction

Chinese hamster ovary (CHO) cells are extensively used to produce biopharmaceuticals^1^ for numerous reasons. While one advantage is their reduced susceptibility to many human virus families^2,3^, there have been episodes of animal viral contamination of biopharmaceutical production runs, mostly from trace levels of viruses in raw materials. These infections have led to expensive decontamination efforts and threatened the supply of critical drugs^4,5^. Viruses that have halted production of valuable therapeutics include RNA viruses such as Cache Valley virus^6^, Epizootic hemorrhagic disease virus^7^, Reovirus^6^ and Vesivirus 2117^8^. Thus, there is a critical need to understand the mechanisms by which CHO cells are infected and how the cells can be universally engineered to enhance their viral resistance^9^. For example, a strategy was proposed to inhibit infection of CHO cells by minute virus of mice by engineering glycosylation^10^. We present an alternative strategy to prevent infections of a number of RNA viruses with different genomic structures and strategies to interfere with the host anti-viral defense.

Many studies have investigated the cellular response to diverse viruses in mammalian cells, and detailed the innate immune responses that are activated upon infection. For example, type I interferon (IFN) responses regulate the innate immune response, inhibit viral infection^11,12^ and can be induced by treatment of cells with poly I:C^13,14^. However, the detailed mechanisms of virus infection and the antiviral response in CHO cells remain largely unknown. Understanding the role of type I IFN-mediated innate immune responses in CHO cells could be invaluable for developing effective virus-resistant CHO bioprocesses. Fortunately, recent genome sequencing^15–17^ and RNA-Seq tools have enabled the analysis of complicated cellular processes in CHO cells^18,19^, such as virus infection.

To unravel the response of CHO cells to viral infection, we infected CHO-K1 cells with RNA viruses from diverse virus families. The RNA viruses are of particular interest since viral RNAs are all sensed by the RIG-I/TLR3 receptor, so broadly active resistance strategies might be engineered upon targeting relevant downstream pathways. We assayed the ability of activators of type I IFN pathways to induce an antiviral response in the cells. Specifically, we asked the following questions: (1) Can CHO-K1 cells mount a robust type I IFN response when infected by RNA viruses? (2) Can innate immune modulators trigger a type I IFN response of CHO-K1 cells and, if so, are the type I IFN levels produced sufficient to protect CHO-K1 cells from RNA virus infections? (3) Which biological pathways and processes are activated during virus infection and/or treatment with innate immune modulators, and are there common upstream regulators that govern the antiviral response? (4) Upon the identification of common upstream regulators, how can we engineer virus resistance into CHO cells for mitigating risk in mammalian bioprocessing? Here we address these questions, illuminate antiviral mechanisms of CHO cells, and guide the development of bioprocess treatments and cell engineering efforts to make CHO cells more resistant to viral infection.

## Materials and Methods

### CHO-K1 cells and RNA virus infections

The susceptibility of CHO-K1 cells to viral infection has been previously reported^3^. Since infectivity was demonstrated for viruses of a variety of families (harboring distinct genomic structures), we selected the following RNA viruses from three different families to be used as prototypes: Vesicular stomatitis virus (VSV, ATCC® VR-1238), Encephalomyocarditis virus (EMCV, ATCC® VR-129B), and Reovirus-3 virus (Reo-3, ATCC® VR-824). Viral stocks were generated in susceptible Vero cells as per standard practices using DMEM (Dulbecco’s Modified Eagle’s medium) supplemented with 10% FBS, 2 mM L-glutamine, 100 U/ml penicillin and 100 µg/ml streptomycin (DMEM-10). Viral stocks were titered by tissue culture infectious dose 50 (TCID_50_) on CHO-K1 cells and used to calculate the multiplicity of infection in the experiments (Table 1).

**Table 1:** Study prototype viruses and multiplicity of infection (MOI) on CHO-K1 cells.

Virus	Virus family	Genomic nucleic acid nature	Referenced CHO cell culture infection	MOI
Vesicular stomatitis virus (VSV)	Rabdoviridae	ss (−) RNA	Potts, 2008	0.003
Encephalomyocarditis virus (EMCV)	Picornaviridae	ss (+) RNA	Potts, 2008	0.007
Reovirus 3 (Reo-3)	Reoviridae	ds RNA	Wisher, 2005; Rabenau 1993	0.0013

#### Virus infection procedures

Cells were seeded in cell culture plates (3 × 10^5^ and 1.2 × 10^6^ cells/well in 96-well and 6-well plates, respectively) and grown overnight in RPMI-1040 supplemented with 10% FBS, 2 mM L-glutamine, 100 U/ml penicillin and 100 µg/ml streptomycin, 10 mM Hepes, 1x non-essential amino acids and 1 mM sodium pyruvate (RPMI-10). IFNα/β (human IFNα (Roferon) and IFNβ (Avonex), mouse IFNα (Bei Resources, Manassas, VA)) as well as innate immune modulators (LPS (TLR4) (Calbiochem), CpG-oligodeoxynucleotide (ODN) D-ODN, 5′-GGTGCATCGATGCAGGGGG-3′^20^ and ODN-1555, 5′-GCTAGACGTTAGCGT-3′ (TLR9) (custom-synthesized at the Center for Biologics Evaluation and Research facility, FDA), imidazoquinoline R837 (TLR7/8) (Sigma) and poly I:C-Low molecular weight/LyoVec (poly I:C) (Invivogen) were added to the cultures 24 h prior to testing or virus infection, at the concentrations indicated in the figures. Note that, by monitoring changes in the gene expression levels of IFNβ and Mx1 in the cells, we established that 16–20 h would be an adequate time interval for treating cells with poly I:C prior to infection (Supplementary Fig. S1). Anti-IFNβ neutralizing antibody (2.5 μg/ml; Abcam, Cambridge, MA cat# 186669) was also used in certain experiments, 24 h prior to infection. Viral infection was performed by adding virus suspensions to the cell monolayers at the indicated MOI in RPMI medium without serum and incubated at 37 °C, 5% CO_2_ for 2 h. Cell cultures were washed twice with 1x PBS to discard unbound virus and further incubated at 37 °C in RPMI-10 for 30 h (VSV), 54 h (EMCV) or 78 h (Reo-3) (unless otherwise indicated in the figures). The cell harvesting time was established based on appearance of cytopathic effect in approximately 50% of the cell monolayer. Cytopathic effect was visualized by crystal violet staining as per standard practices. We used 50% cytopathic effect to be end point in determining CHO cell viral susceptibility as a way to “standardize” the effect of the three viruses over the host cell and look at transcriptome changes at a time in which the culture was similarly affected. Since the quantification of the response would be provided by the RNA-Seq data, we adopt the qualitative approach to assess the susceptibility of the cells to virus infection. Infection/poly I:C experiments were repeated twice, independently. In each experiment, CHO cells were cultured as poly I:C untreated – uninfected (media control, m), poly I:C treated – uninfected (p), poly I:C untreated – virus infected (Vm) and poly I:C treated – virus infected (Vp).

#### Western blot procedures

Cell lysates were prepared using mammalian protein extraction reagent M-PER (Thermo Fisher Scientific, Waltham, MA) with Protease and Halt™ phosphatase inhibitor cocktails (Thermo Fisher Scientific) using an equal number of cells per sample. Samples were analyzed by SDS-PAGE using 10–20% Tris-Glycine gels (Thermo Fisher Scientific) under reducing conditions. As a molecular weight marker, protein ladder (cat# 7727S) from Cell Signaling Technology (Danvers, MA) was used. Nitrocellulose membranes and iBlot™ transfer system (Thermo Fisher Scientific) were used for Western Blot analysis. All other reagents for Western Blot analyses were purchased from Thermo Fisher Scientific. Membranes were blocked with nonfat dry milk (BIO-RAD, Hercules, CA) for 1 h followed by incubation with primary antibodies against STAT1, pSTAT1 (pY701, BD Transduction Lab, San Jose, CA), or Mx1 (gift from O. Haller, University of Freiburg, Freiburg, Germany) O/N at 4 °C. Secondary goat anti-mouse and anti-rabbit antibodies were purchased from Santa Cruz Biotechnology. SuperSignal West Femto Maximum Sensitivity Kit (Thermo Fisher Scientific) was used to develop membranes, and images were taken using LAS-3000 Imaging system (GE Healthcare Bio-Sciences, Pittsburgh, PA).

### RNA extraction, purification, and real-time PCR

Cell cultures were re-suspended in RLT buffer (Qiagen) and kept at −80 °C until RNA was extracted using the RNeasy kit (Qiagen) and on-column DNAse digestion. RNA was eluted in 25 µl of DEPC water (RNAse/DNAse free); concentration and purity were tested by bioanalyzer. Total RNA levels for type I IFN related genes and viral genome were also assessed by RT-PCR. Complementary DNA synthesis was obtained from 1 μg of RNA using the High capacity cDNA RT kit (Thermo Fisher scientific) as per manufacturer’s instructions. Semi-quantitative PCR reactions (25 µl) consisted in 1/20 cDNA reaction volume, 1x Power Sybr master mix (Thermo Fisher Scientific), 0.5 µM Chinese hamster-specific primers for IFNβ, Mx1, IRF7 and IITMP3 sequences (SAbiosciences). Eukaryotic 18S was used as a housekeeping gene and assessed in 1X Universal master mix, 18S expression assay (1:20) (Applied Biosystems) using a 1/50 cDNA reaction volume. Fold changes were calculated by the 2-ΔΔCt method. Note that, to be consistent, this is a similar qualitative approach to assess the activation of the host cell innate immune response to the virus or poly I:C, in which the quantification of the response would be provided by the RNA-Seq data.

### cDNA library construction and Next-generation sequencing (RNA-Seq)

Library preparation was performed with Illumina’s TruSeq Stranded mRNA Library Prep Kit High Throughput (Catalog ID: RS-122-2103), according to manufacturer’s protocol. Final RNA libraries were first quantified by Qubit HS and then QC on Fragment Analyzer (from Advanced Analytical). Final pool of libraries was run on the NextSeq platform with high output flow cell configuration (NextSeq® 500/550 High Output Kit v2 (300 cycles) FC-404-2004). Raw data are deposited at the Gene Expression Omnibus and Short Read Archive (accession numbers: GSE119379).

### RNA-Seq quantification and differential gene expression analysis

RNA-Seq quality was assessed using FastQC. Adapter sequences and low-quality bases were trimmed using Trimmomatic^21^. Sequence alignment was accomplished using STAR^22^ against the CHO genome (GCF_000419365.1_C_griseus_v1.0) with default parameters. HTSeq^23^ was used to quantify the expression of each gene. We performed differential gene expression analysis using DESeq2^24^. After Benjamini-Hochberg FDR correction, genes with adjusted p-values less than 0.05 and fold change greater than 1.5 were considered as differentially expressed genes (DEGs). Supplementary Table S1 shows the number of identified DEGs in the three different comparisons: 1) untreated – uninfected vs. untreated – virus infected (m vs. Vm); 2) untreated – uninfected vs. poly I:C treated – uninfected (m vs. p); and 3) untreated – virus infected vs. poly I:C treated – virus infected (Vm vs. Vp).

### Genetic engineering (Gfi1, Trim24, Gfi1/Trim24) of CHO-S cell lines

CHO-S cells (Thermo Fisher Scientific Cat. # A1155701) and KO clones were cultured in CD CHO medium supplemented with 8 mM L-glutamine and 2 mL/L of anti-clumping agent (CHO medium) in an incubator at 37 °C, 5% CO_2_, 95% humidity. Cells were transfected using FuGENE HD reagent (Promega Cat. # E2311). The day prior to transfection, viable cell density was adjusted to 8 × 10^5^ cells/mL in an MD6 plate well containing 3 mL CD CHO medium supplemented with 8 mM L-glutamine. For each transfection, 1500 ng Cas9-2A-GFP plasmid and 1500 ng gRNA plasmid (see Text S1 for details about the construction of plasmids) were diluted in 75 uL OptiPro SFM. Separately, 9 uL FuGene HD reagent was diluted in 66 uL OptiPro SFM. The diluted plasmid was added to the diluted FuGENE HD and incubated at room temperature for 5 minutes and the resultant 150 µL DNA/lipid mixture was added dropwise to the cells. For viability experiments, CHO-S KO cell lines were seeded at 3 × 10^6^ cells in 30 ml in CHO medium and incubated at 37 °C, 5% CO_2_, 125 rpm for up to 7 days. Infections were conducted with EMCV and Reo-3 at the same MOI calculated in CHO-K1 cells for 2 h prior to wash cells twice to discard unbound particles. Control cell lines showing susceptibility to either virus were infected in parallel to those with Gfi1 and Trim24 gene KO.

### Single cell sorting, clone genotyping and expansion

Transfected cells were single cell sorted 48 hours post transfection, using a FACSJazz, based on green fluorescence with gating determined by comparison to non-transfected cells. Sorting was done into MD384 well plates (Corning Cat. # 3542) containing 30 µL CD CHO medium supplemented with 8 mM L-glutamine, 1% antibiotic-antimycotic agent (Thermo Fisher Scientific Cat. # 15240-062) and 1.5% HEPES buffer (Thermo Fisher Scientific Cat. # 15630-056). After 15 days, colonies were transferred to an MD96F well plate (Falcon Cat. # 351172) containing 200 µL CD CHO medium supplemented with 8 mM L-glutamine, and 1% antibiotic-antimycotic. After additional two days, 50 µL cell suspension from each well was transferred to a MicroAmp Fast 96 well reaction plate (Thermo Fisher Scientific Cat. # 4346907), along with 5 × 10^5^ wildtype control cells. The plate was centrifuged at 1000 x g for 10 minutes and then the supernatant was removed by rapid inversion. Twenty µL of 65 °C QuickExtract DNA Extraction Solution (Epicentre Cat. # QE09050) was added to each well and mixed. The plate was then placed in a thermocycler at 65 °C for 15 minutes followed by 95 °C for 5 minutes. Amplicons were generated for each gene of interest per well using Phusion Hot Start II DNA Polymerase and verified to be present visually on a 2% agarose gel. Amplicons from each well had unique barcodes, allowing them to be pooled and purified using AMPure XP beads (Beckman Coulter Cat. # A63881) according to manufacturer’s protocol, except using 80% ethanol for washing steps and 40 µL beads for 50 µL sample. Samples were indexed using the Nextera XT Index kit attached using 2 x KAPA HiFi Hot Start Ready mix (Fisher Scientific Cat. # KK2602). AMPure XP beads were used to purify the resulting PCR products. DNA concentrations were determined with the Qubit 2.0 Fluorometer and used to pool all indices to an equimolar value and diluted to a final concentration of 10 nM using 10 mM Tris pH 8.5, 0.1% Tween 20. The average size of the final library was verified with a Bioanalyzer 2100. The amplicon library was then sequenced on an Illumina MiSeq. Insertions and deletions were identified by comparison of expected versus actual amplicon size. Clones with frameshift indels in all alleles were selected for expansion in shake flasks (shaking at 120 rpm, 25 mm throw), banking and characterization.

## Results and Discussion

### CHO-K1 cells fail to resolve infection by RNA viruses despite possessing functional type I IFN-inducible anti-viral mechanisms

**Figure 1:**
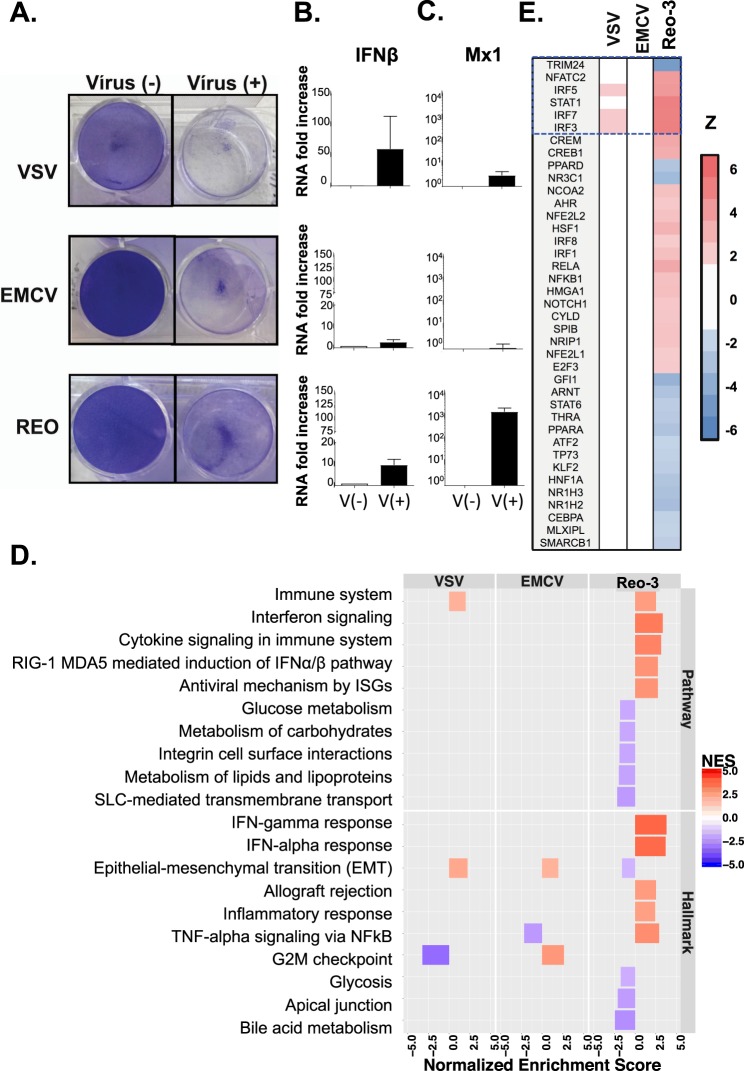
RNA viruses induce cytopathic effects on CHO-K1 cells. (**A**) Cytopathic effect of the three RNA viruses on CHO cells upon 30 h (VSV), 54 h (EMCV) or 78 h (Reo-3) of infection. Fold change in IFNβ (**B**) and Mx1 (**C**) gene expressions in CHO cells infected with the three RNA viruses compared to uninfected cells at the same time points. (**D**) Several pathways and processes were enriched for differentially expressed genes following viral infection (m vs. Vm). (**E**) Top activated (red) or repressed (blue) upstream regulators following virus infection.

To evaluate the response of CHO cells to three different RNA viruses (VSV, EMCV and Reo-3; see Table 1), cells were infected and monitored for cytopathic effects and gene expression changes related to the type I IFN response. All three viruses induced a cytopathic effect (Fig. 1A, right panels) and a modest increase in IFNβ transcript levels in infected CHO cell cultures was measured (Fig. 1B), suggesting limited production of IFN. Through its cellular receptor, IFNα/β can further activate downstream interferon-stimulated genes known to limit viral infection both in cell culture and *in vivo*^25,26^. We noted that CHO cells seem to have a functional IFNα/β receptor and its activation with exogenous IFN confers resistance of CHO cells to VSV infection (see Supplementary Text S2 and Fig. S2). Interestingly, CHO cells expressed high levels of the antiviral gene Mx1 when infected with Reo-3, but not VSV and EMCV (Fig. 1C). Nevertheless, the virus-induced IFN mRNA response in the host cell was insufficient to prevent cell culture destruction. These data suggest a possible inhibition of the antiviral type I IFN response that varies across viruses, as previously reported^27,28^.

To explore why the induced type I IFN failed to mount a productive antiviral response in CHO cells, we conducted RNA-Seq and pathway analysis using GSEA (see details in Supplementary Text S3 and Table S1). GSEA analysis that compared control vs. infected CHO cells (m vs. Vm) revealed the modulation of several immune-related gene sets and pathways activated by the virus (Fig. 1D and Supplementary Fig. S3, Table S2, and Text S4). Unlike VSV and EMCV, Reo-3 induced the ‘interferon alpha response’ and ‘RIG-I and MDA5-mediated induction of IFNα’ pathways ((p-value, NES) = (9.05 × 10^−3^, 3.68) and (1.12 × 10^−2^, 2.74), respectively). These findings were consistent with observations that the reovirus genome (dsRNA) can stimulate TLR3 and RIG-I to induce innate immune responses in other cell types^29,30^, in which the observed responses diverged markedly from the VSV and EMCV infections.

As we observed for Mx1, only Reo-3-infected cells showed a significant enrichment of differentially expressed genes involved in the type I IFN response (FDR-adjusted p-value = 9.05 × 10^−3^; normalized enrichment score, NES = 3.68). These genes contain the consensus transcription factor binding sites in the promoters that are mainly regulated by the transcription factor STAT1 and the interferon regulatory factors (IRF) family, such as IRF1, IRF3, IRF7 and IRF8 (Fig. 1E). These results are consistent with observations that the IRF family transcription factors activate downstream immune responses in virus-infected mammalian cells^31,32^. In contrast, VSV and EMCV failed to trigger anti-viral related mechanisms (e.g., type I IFN responses) downstream of IFNβ (Figs 1D and S3A). Examples of a few pathways that were stimulated included ‘immune system’ (including adaptive/innate immune system and cytokine signaling in immune system) in VSV (FDR-adjusted p-value = 1.49 × 10^−2^; normalized enrichment score, NES = 1.99) and the ‘G2M checkpoint’ in EMCV (p-value = 8.95 × 10^−3^; NES = 2.64). Disruption of the cell cycle affecting the G2M DNA checkpoint network has been reported for the survival of several viruses, including HIV (ssRNA)^33^, EBV (dsDNA)^34^, JCV (DNA)^35^, HSV (DNA)^36^. However, further studies will need to confirm whether VSV or EMCV use a similar strategy to escape the cell defense. Nevertheless, neither VSV nor EMCV infection activated known upstream activators of type I IFN pathways (Fig. 1E) when analyzed with Ingenuity Pathway Analysis (IPA)^37^.

### Poly I:C induces a robust type I interferon response in CHO cells

Type I IFN responses limit viral infection^11,12,38^, and innate immune modulators^39,40^ mimic pathogenic signals and stimulate pattern recognition receptors (PRRs), leading to the activation of downstream immune-related pathways. Intracellular PRRs, including toll-like receptors (TLR) 7, 8 and 9, and cytosolic receptors RIG-I or MDA5, can sense viral nucleic acids and trigger the production of type I IFN. Thus, we asked whether CHO cell viral resistance could be improved by innate immune modulators.

**Figure 2:**
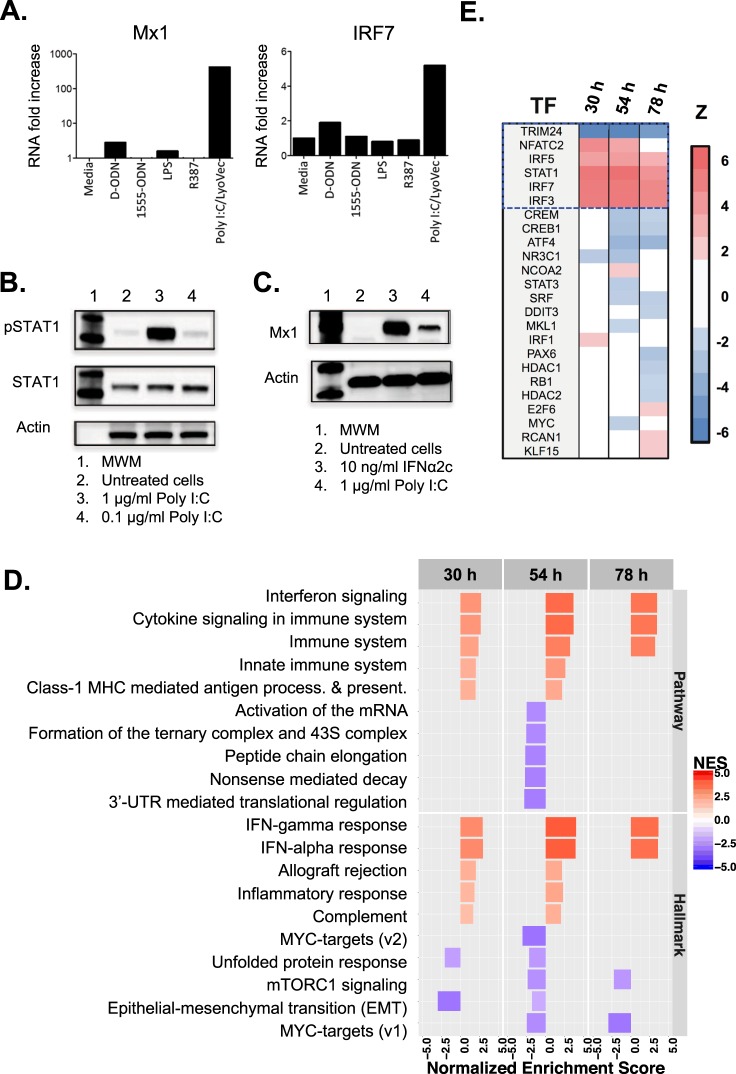
Innate immunity genes in CHO cells are activated by poly I:C. (**A**) IFN-stimulated transcription was increased in cells treated with poly I:C /LyoVec for 24 h, but not with other TLR ligands engaging TLR9, TLR4 or TLR7/8. (**B**) Poly I:C triggered STAT1 phosphorylation when used at 1 g/L, and (**C**) the levels of Mx1 protein expression were comparable to those triggered by IFNα2c. Note that, the antibodies used here and the assay procedures are detailed in the Methods section. (**D**) Several pathways and processes were enriched for differentially expressed genes following poly I:C treatment (m vs. p). (**E**) Top upstream regulators that are activated (red) or repressed (blue) following poly I:C treatment. All full-length blots are presented in the Supplementary Fig. S12.

CHO PRRs have not been studied extensively, so we first assessed the ability of synthetic ligands to stimulate their cognate receptors to induce a type I IFN response. CHO cells were incubated with LPS (TLR4 ligand), CpG-oligodeoxynucleotide (ODN) type D (activates TLR9 on human cells), ODN-1555 (activates TLR9 on murine cells), imidazoquinoline R837 (TLR7/8 ligand) and poly I:C-Low molecular weight/LyoVec (poly I:C) (activates the RIG-I/MDA-5 pathway), and subsequently tested for changes in expression of IFN stimulated genes with anti-viral properties. After 24 h of culture, gene expression levels of IRF7 and Mx1 increased significantly in cells treated with poly I:C but not in those treated with any of the other innate immune modulators (Fig. 2A). Furthermore, STAT1 phosphorylation and Mx1 protein levels were elevated following treatment with poly I:C or exogenous interferon-alpha (IFNα), which was used as a control (Fig. 2B,C).

Next, we characterized the type I IFN response induced by poly I:C by analyzing the transcriptome of untreated vs. treated CHO cells. Cells were cultured with poly I:C in the media for 30, 54 and 78 h after an initial 16 h pre-incubation period (see Methods for details). GSEA of the RNA-Seq data demonstrated that poly I:C induced a strong ‘innate immune response’ in comparison to untreated cultures (media) (m vs. p; (p-value, NES, Enrichment strength) = (8.08 × 10^−3^, 2.98, 73%), (1.57 × 10^−2^, 3.95, 70%) and (3.91 × 10^−3^, 3.58, 78%)) evident in the three independently tested time points (Fig. 2D and Supplementary Fig. S3B, Text S4 and Table S3). In addition, poly I:C activated several upstream regulators of the type I IFN pathways (Fig. 2E). We note that the GSEA strength (see Supplementary Text S3) of the innate immune response induced by poly I:C (m vs. p) was stronger than the innate immune response seen for Reo-3 infection alone (m vs. Vm in Supplementary Fig. S3). Thus, CHO cells can activate the type I IFN signaling (JAK-STAT) pathway in response to poly I:C and display an anti-viral gene signature, which was sustained for at least 4 days.

### Poly I:C-induced type I interferon response protects CHO cells from RNA virus infections

**Figure 3:**
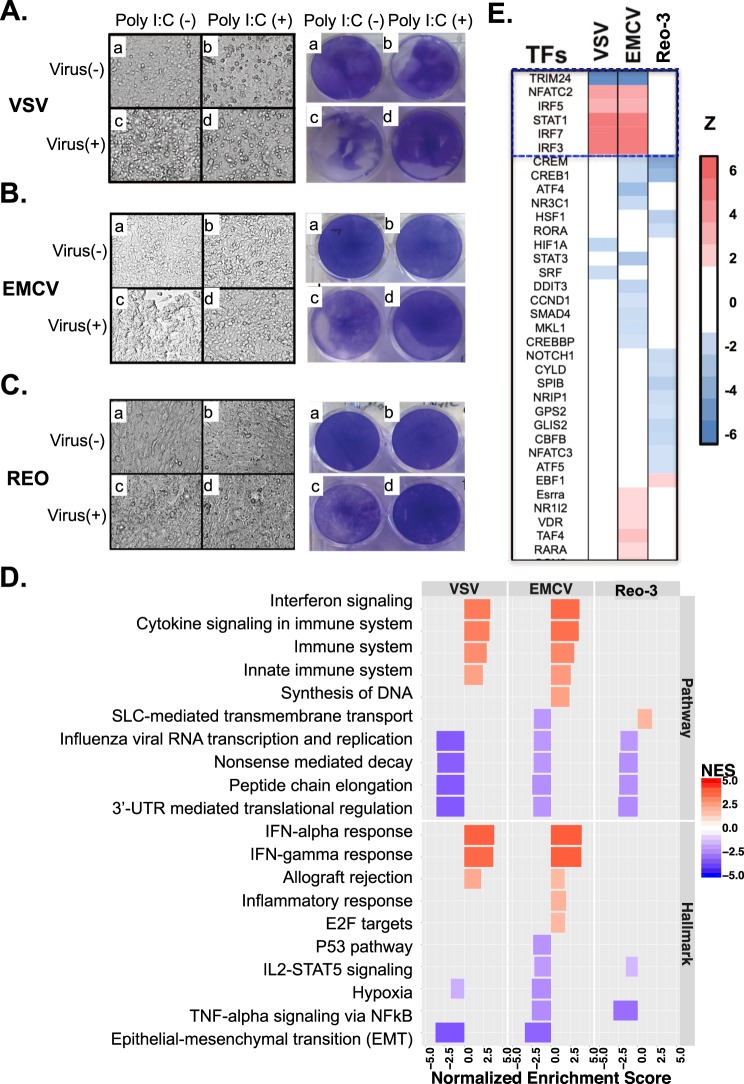
Poly I:C pre-treatment prevents virus infection of VCV, EMCV, and Reo-3. (**A**–**C**) Cell morphology (left panels) and cytopathic effect measured by crystal violet staining (right panels) of virus-infected CHO cells; (**D**) The enriched down-stream pathways under condition of Vm vs. Vp using RNA-Seq data. (**E**) The top 35 upstream regulators that are activated or repressed by poly I:C pre-treatment. A full list of the activated or repressed upstream regulators is shown in the Table S5.

We next examined if the type I IFN response, induced by poly I:C, could protect CHO cells from RNA virus infections. We found that poly I:C pre-treatment protected CHO cells against VSV infection through the IFNβ-mediated pathway (Supplementary Fig. S4 and Text S5), and that poly I:C protected against all three viruses tested (Fig. 3A–C). Cell morphology differed notably between cultures infected with virus (Vm), control uninfected cells (m), and poly I:C pre-treated cultures (p and Vp) (Fig. 3A–C, left panels). These morphological changes correlated with the cytopathic effect observed in the cell monolayers (Fig. 3A–C, right panels). At 78 h, the extent of cell culture damage by Reo-3, however, was milder than by VSV and EMCV at a shorter incubation times (30 h and 54 h, respectively) (Panels Vm in Fig. 3A–C), possibly since Reo-3 induced higher levels of anti-viral related genes in the CHO cells but VSV and EMCV did not (Fig. 1C–E). Notably, although poly I:C pre-treatment conferred protection of CHO cells to all three viral infections (Panels Vp in the Fig. 3A–C), striking transcriptomic differences were observed (Supplementary Table S4). Poly I:C pre-treatment significantly activated immune-related pathways and up-regulated type I IFN-related gene expression in CHO cells infected with VSV and EMCV when compared to non-poly I:C pre-treated cells that were infected (Vm vs. Vp) (Fig. 3D,E, Supplementary Fig. S5A,B and Table S5). Poly I:C pre-treatment was sufficient to induce a protective type I IFN response to VSV and EMCV. In contrast, for Reo-3 infection, pre-treatment with poly I:C did not further increase the levels of expression of IFN associated genes already observed in no pre-treated cells. The lack of enhanced expression of antiviral genes in Reo-3 Vm vs. Vp observed in the GSEA was further confirmed by Taqman analysis. A similar level of expression of anti-viral Mx1 and IITMP3 genes^41–44^ was obtained for CHO cells independently infected with Reo-3 (Vm), treated with poly I:C (p), or pre-treated with poly I:C and infected (Vp), which resulted in no differences in transcript levels when we compared Vm vs. Vp (Supplementary Fig. S5C). Nevertheless, the outcome of infection was surprisingly different in Vm or Vp samples. To understand these differences, we searched for genes that were differently modulated by poly I:C treatment in the context of Reo-3 infection. Indeed, we identified 30 genes (Supplementary Fig. S6 and Table S6) that were significantly up regulated (adjusted p-value < 0.05, fold change > 1.5) in the comparisons of m vs. Vp and m vs. p but not in the comparison of m vs. Vm. These genes are significantly enriched in 11 KEGG pathways (https://www.kegg.jp/kegg/) related to host-immune response (e.g., antigen processing and presentation, p-value = 3.4 × 10^−3^) and processes important to virus infection (e.g., endocytosis, p-value = 2.5 × 10^−2^). We also observed many of these genes significantly enriched molecular functions: 1) RNA polymerase II transcription factor activity (11 genes; GO:0000981 FDR-adjusted p-value < 1.30 × 10^−15^) and 2) nucleic acid binding transcription factor activity (12 genes GO:0001071 FDR-adjusted p-value < 3.54 × 10^−15^) by gene set enrichment analysis (see Supplementary Text S3 and Table S7). This suggests that poly I:C treatment, 16 hours prior to virus infection, pre-disposes the cell to an antiviral state and might restore the host transcription machinery subverted by Reo-3 virus, resulting in the protection of the CHO cells. Further experiments would be interesting to investigate whether these identified molecular functions using transcriptomic data could directly contribute to protect CHO cells from Reo-3 virus infection.

Our results revealed other processes that are differentially activated or repressed between Vm and Vp (Fig. 3D and Supplementary Table S4). For example, the top down-regulated Reactome pathways in the virus-infected cells (Vm vs. Vp) are protein translational related processes: ‘nonsense mediated decay enhanced by the exon junction complex’ (p-value = 3.32 × 10^−2^, NES = −3.50), ‘peptide chain elongation’ (p-value = 3.32 × 10^−2^, NES = −3.59), and ‘3′-UTR mediated translational regulation’ (p-value = 3.38 × 10^−2^, NES = −3.61). These results agree with studies showing viral hijacking of the host protein translation machinery during infection^45^, and that the activation of interferon-stimulated genes restrain virus infections by inhibiting viral transcription and/or translation^38^. All these results suggest that poly I:C treatment provides the cell with an advantageous immune state that counteracts viral escape mechanisms and results in cell survival.

### A STAT1-dependent regulatory network governs viral resistance in CHO cells

GSEA revealed that several transcriptional regulators were activated or repressed during different viral infections and poly I:C-treated cells (Figs 1E, 2E and 3E). Among these, NFATC2, STAT1, IRF3, IRF5, and IRF7 were consistently activated by poly I:C pre-treatment of CHO cells (m vs. p and Vm vs. Vp), and TRIM24 was suppressed. These transcription factors are involved in TLR-signaling (IRF3, IRF5, and IRF7)^31^ and JAK/STAT signaling (NFATC2, STAT1, and TRIM24). The TLR signaling pathway is a downstream mediator in virus recognition/response and in activating downstream type-I interferon immune responses^46,47^. Meanwhile, the JAK/STAT pathway contributes to the antiviral responses by up-regulating interferon simulated genes to rapidly eliminate virus within infected cells^48–50^. Importantly, one mechanism by which STAT1 expression and activity may be enhanced is via the poly I:C-induced repression of TRIM24 (an inhibitor of STAT1). The crosstalk between TLR- and JAK/STAT-signaling pathways is therefore important in virus clearance of infected host cells^51^.

**Figure 4:**
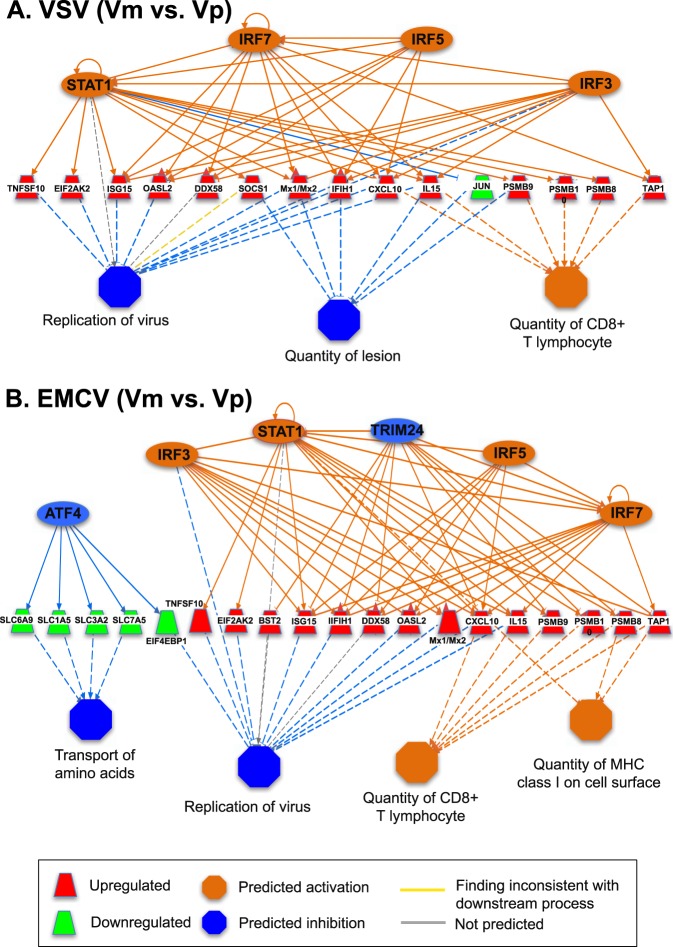
A STAT1-dependent regulatory network controls viral resistance (VSV and EMCV) in CHO cells. A STAT1-dependent regulatory network induced by the pre-treatment of poly I:C leads to the inhibition of VSV (**A**) and EMCV (**B**) replication in CHO cells, based on the comparison of Vm and Vp RNA-Seq. The colors denote the states inferred from the RNA-Seq data. For example, the blue color of TRIM24 means that TRIM24 activity is suppressed, based on the differential expression of genes that are regulated by TRIM24.

To better understand the role of upstream regulators in the CHO cell viral protection, we examined the expression of the affected downstream target genes. Tables 2 and 3 show the regulatory pathways modulated by poly I:C treatment in uninfected (m vs. p; Table 2) or infected (Vm vs. Vp; Table 3) cells, and the described downstream effect. In cells surviving VSV and EMCV infection (Vp), we identified regulatory networks involved in restricting viral replication (Table 3 and Fig. 4A,B). These networks are predominantly regulated by the 6 transcription factors (NFATC2, STAT1, IRF3, IRF5, IRF7, and TRIM24) that were also identified as transcription factors induced in poly I:C treated uninfected cells (p) (Table 2). These findings suggest that the induction of the STAT1-dependent regulatory network by poly I:C treatment allows the cell to adopt an activated state that makes it refractory to virus infection. In contrast, the STAT1-dependent regulatory network was not apparent when comparing Reo-3 infected cells untreated and treated with poly I:C (Vm vs. Vp), because both Reo-3 and poly I:C induce STAT1 in CHO cells (Figs 1E and 2E). Poly I:C is a structural analog of double-stranded RNA and activates similar pathways as Reo-3^52^, such as, the NFATC2-dependent (Supplementary Fig. S7) and IRF3-dependent networks (Supplementary Fig. S8).

**Table 2:** The downstream effects of the upstream regulators from the comparison of m vs. p.

Virus	Consis-tency score^*a^	Total nodes (TF, TG, BP)	Transcription factors (TF)^*b^	Target gene (TG)^*c^	Biological Process (BP)^*d^	Relations^*e^
30 h	5.82	21 (5, 13, 3)	**STAT1**, IRF3, IRF5, IRF7, NFATC2	CASP1, CXCL10, DDX58, EIF2AK2, IFIH1, IL15, ISG15, Mx1/Mx2, OASL2, PELI1, PML, SOCS1, TNFSF10	**Inhibit** Replication of virus. **Activate** Activation of phagocytes; Apoptosis of antigen presenting cells.	6/15 (40%)
54 h	22.47	48 (7.29.12)	**STAT1**, IRF3, IRF5, IRF7, NFATC2, TRIM24, NCOA2	BST2, C3, CASP1, CXCL10, DDX58, EGR2, EIF2AK2, GBP2, IFIH1, IFIT1B, IFIT2, IFITM3 (IITMP3), Igtp, IL15, ISG15, Mx1/Mx2, MYC, OASL2, PML, PSMB10, PSMB8, PSME2, PTGS2, SPP1, STAT2, TAP1, TLR3, TNFSF10, TRAFD1	**Inhibit** Replication of virus; Infection by RNA virus; Infection of central nervous system. **Activate** Antiviral response; Clearance of virus; Immune response of antigen presenting cells; Immune response of phagocytes; Cytotoxicity of leukocytes; Function of leukocytes; Infiltration by T lymphocytes; Quantity of MHC Class I of cell surface; Cell death of myeloid cells.	21/84 (25%)
78 h	27.80	30 (8, 14, 8)	**STAT1**, IRF5, NFATC2, NR3C1, PPARD, ZBTB16, CDKN2A, EBF1	C3, CCL2, CCL7, CD36, CXCL10, CXCL9, DDX58, EIF2AK2, ISG15, MYC, THBS1, TLR3, TNFSF10, VEGFA	**Activate** Activation of macrophages; Apoptosis of myeloid cells; Cell movement of T lymphocytes; Cellular infiltration by leukocytes; Damage of lung; Recruitment of leukocytes; Response of myeloid cells; Response of phagocytes.	11/64 (17%)
78 h	7.56	12 (2, 7, 3)	CDKN2A, ZBTB16	C3, CCL2, CCL7, CXCL10, CXCL9, MYC, VEGFA	**Activate** Cell movement of T lymphocytes; Recruitment of leukocytes; Survival of organism.	1/6 (17%)

*^a^Consistency score is to measure the consistency of a predicted network by IPA with the literature evidences.

*^b,c^The upstream regulators (STAT1 is highlighted in bold face) and the antiviral relating genes.

*^d^The biological functions known to associated with the regulatory networks annotated by the IPA.

*^e^The number of identified relationships and the total relationships that represent the known regulatory relationships between regulators and functions supported by literatures annotated by the IPA.

**Table 3:** The downstream effects of the upstream regulators from the comparison of Vm vs. Vp.

Virus	Consis-tency score	Total nodes (TF, TG, BP)^*a^	Transcription factors (TF) ^*b^	Target genes (TG)^*c^	Biological Process (BP)^*d^	Relations^*e^
VSV	8.00	22 (4, 15, 3)	**STAT1**, IRF3, IRF5, IRF7	CXCL10, DDX58, EIF2AK2, IFIH1, IL15, ISG15, JUN, Mx1/Mx2, OASL2, PSMB10, PSMB8, PSMB9, SOCS1, TAP1, TNFSF10	**Inhibit** Replication of virus; Quantity of lesion. **Activate** Quantity of CD8+ T lymphocyte.	2/12 (17%)
EMCV	12.16	29 (6, 19, 4)	**STAT1**, IRF3, IRF5, IRF7, TRIM24, ATF4	BST2, CXCL10, DDX58, EIF2AK2, EIF4EBP1, IFIH1, IL15, ISG15, Mx1/Mx2, OASL2, PSMB10, PSMB8, PSMB9, SLC1A5, SLC3A2, SLC6A9, SLC7A5, TAP1, TNFSF10	**Inhibit** Replication of virus; Transport of amino acids. **Activate** Quantity of CD8+ T lymphocyte; Quantity of MHC Class I on cell surface.	3/24 (13%)
EMCV	7.91	18 (2, 10, 6)	CCND1, SMAD4	AREG, CCND2, EREG, GJA1, HSPA8, ITGAV, NFKBIA, PTGS2, SOX4, SPP1	**Inhibit** Arthritis; Cell cycle progression; Cell viability; Growth of ovarian follicle; Proliferation of cells. **Activate** Edema.	7/12 (58%)
EMCV	6.96	19 (2, 10, 7)	MKL1, VDR	CAMP, CCL2, HLA-A, ICAM1, IL6, MMP9, PTGS2, RELB, SPP1, TNC	**Inhibit** Cancer; Quantity of interleukin; Rheumatic Disease; Development of body trunk. **Activate** Cell death of connective tissue cells; Nephritis; Organismal death.	7/14 (50%)
Reo-3	5.61	21 (4, 14, 3)	GFI1, NR1H3, NRIP1, PPARG	ACACB, CAV1, CD36, CSF3, ETS1, ID2, IL6, LDLR, LPL, NFKBIA, PDK2, PDK4, PPARA, SLC2A1	**Inhibit** Oxidation of carbohydrate; Production of leukocytes; Quantity of vldl triglyceride in blood.	1/12 (8%)

*^a^Consistency score is to measure the consistency of a predicted network by IPA with the literature evidences.

*^b,c^The upstream regulators (STAT1 is highlighted in bold face) and the antiviral relating genes.

*^d^The biological functions known to associated with the regulatory networks annotated by the IPA.

*^e^The number of identified relationships and the total relationships that represent the known regulatory relationships between regulators and functions supported by literatures annotated by the IPA.

### Deletion of Trim24 and Gfi1 induced CHO cell innate immunity and viral resistance

**Figure 5:**
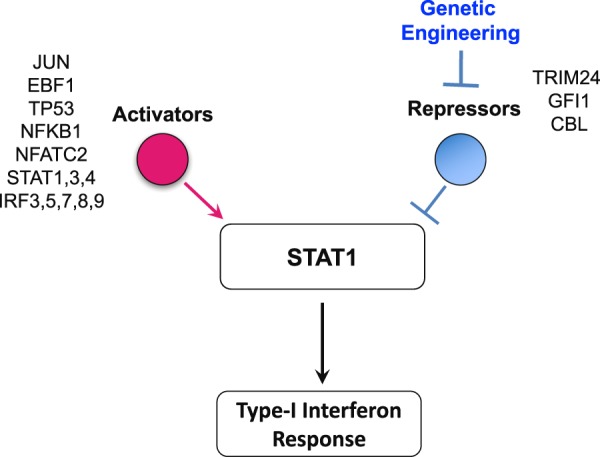
Identification of regulators of STAT1 as candidates for engineering the antiviral response. Schematic of the regulators of STAT1, which may be candidates for engineering and improving virus resistance in CHO cells.

**Figure 6:**
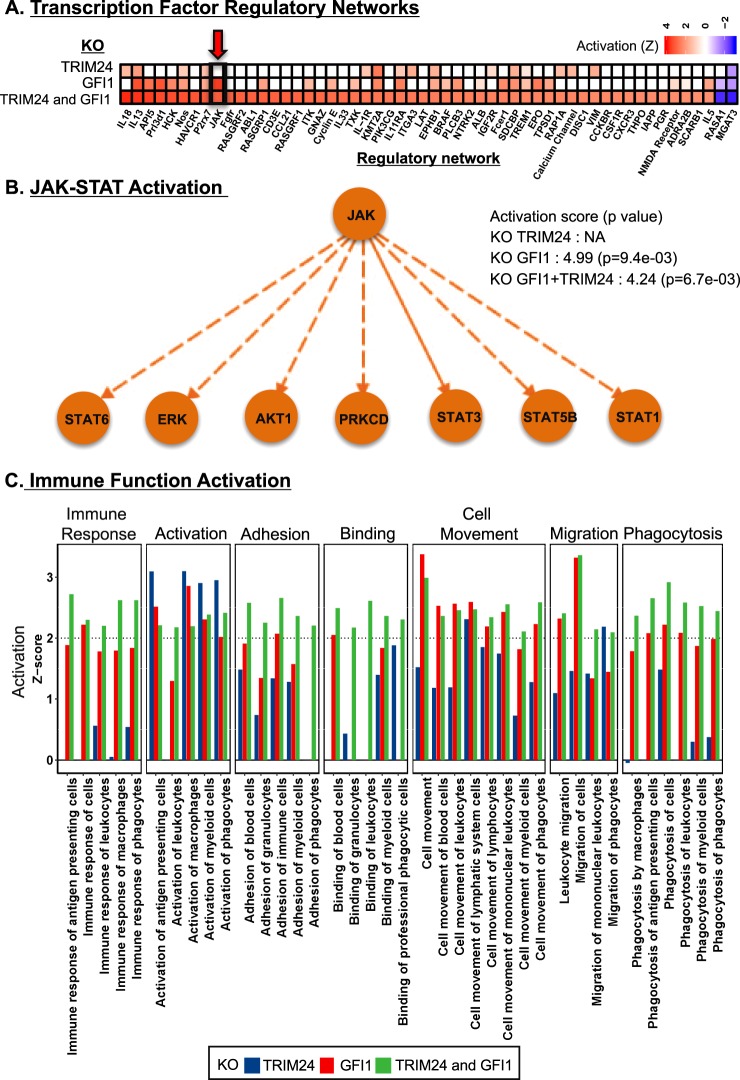
RNA-Seq results of the Gfi1 and/or Trim24 KO engineered CHO cells. Gfi1 and Trim24 were knocked out compared to the control (susceptible) cells. Transcriptional regulatory networks were identified using IPA upstream regulatory analysis (**A**), in which the innate immunity regulatory network (JAK-STAT network) is indicated by the red arrow. Transcriptional factors of the identified JAK-STAT regulatory network in the knocked down cells (**B**) and the activation of immune functions following Gfi1 and/or Trim24 genetic engineering were illustrated (**C**).

**Figure 7:**
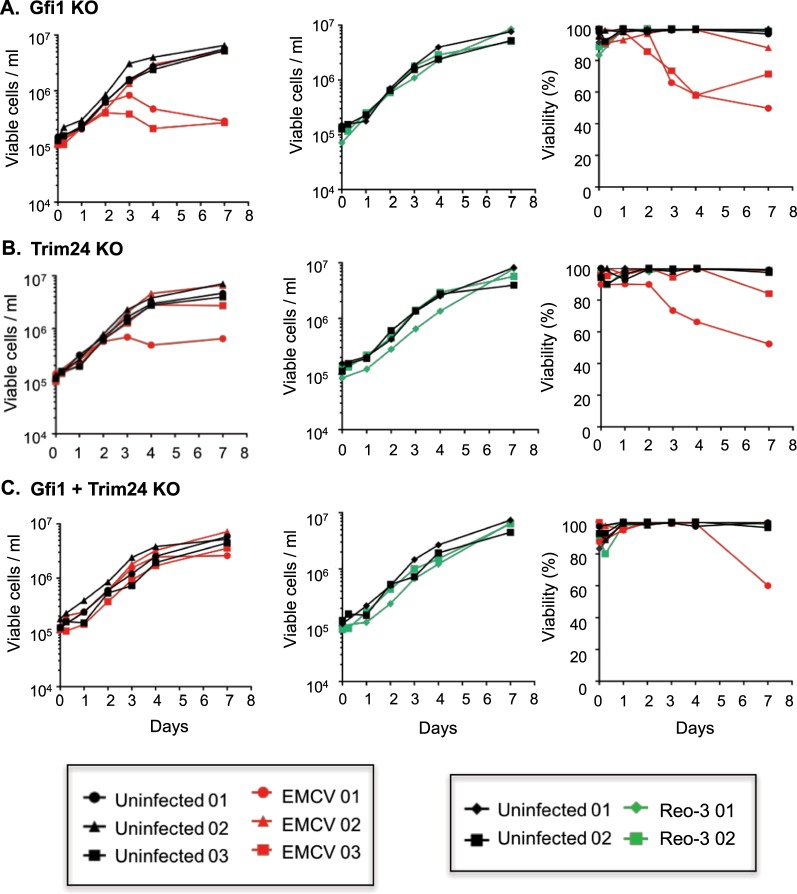
Viral resistance (viable cell density and viability) of the Gfi1 and/or Trim24 KO engineered CHO cells. Gfi1 and Trim24 were knocked out and tested for resistance to EMCV and Reo-3 virus infection compared to the control (susceptible) cells. Cell density and viability was followed up for one week post infection (p.i.) for Gfi1 single knockout cells (**A**), Trim24 single knockout cells (**B**) and Gfi1 and Trim24 double knockout cells (**C**). Data shown is from three (EMCV) and two (Reo-3) independent virus infection experiments. Susceptible CHO cell lines were used as positive controls for EMCV and Reo-3 virus infections during the first seven days (Fig. S10). In some experiments, resistant cultures were passaged and followed up for an additional week (Fig. S11).

With the STAT1 network potentially contributing to viral resistance, we searched for upstream regulators that could be modulated to maximally induce STAT1. We first used the IPA upstream regulator analysis tool to obtain all the predicted upstream regulators (TFs) in the RNA-Seq data (comparisons: m vs. p and Vm vs. Vp). Then, we evaluated these TFs for their potential to regulate the STAT1 gene. Finally, we identified sixteen statistically significant (p < 0.05) upstream regulators, including 13 positive and 3 negative regulators of STAT1 using IPA (Fig. 5; see details in Supplementary Text S6, Fig. S9 and Table S8). We hypothesized that the deletion of the most active repressors of STAT1 could improve virus resistance by inducing STAT1 gene expression and the downstream type I IFN antiviral response in the cell (Fig. 5). We identified three STAT1 repressors (Trim24, Gfi1 and Cbl) with a negative regulatory score and therefore potential for inhibiting STAT1 based on the RNA-Seq differential expression data (see details in Supplementary Text S6 and Fig. S9). However, Cbl was not present in cells infected with Reo-3 (Table S8). Therefore, we selected the two negative regulators, Gfi1^53^ and Trim24^54^ of STAT1 and knocked them out in CHO-S cells using Crispr/Cas9 (see details in the Methods section). To evaluate the impact of gene editing on the engineered CHO-S cells, we conducted RNA-Seq in uninfected single (Gfi1 or Trim 24) or double (Gfi1 + Trim 24) KO cell lines (Fig. 6). Our results revealed that these cells had increased transcript levels of a number of genes involved in innate immunity pathways, such as those mediated by interleukins (ILs) (e.g IL-33 pathway (IL-1R, IL-5, IL-13, IL-33) and IL-18) (Fig. 6A) and STAT (e.g., STAT1, 3, 5B and 6)-related genes (Fig. 6B), leading to the upregulation of several immune functions^55,56^ (Fig. 6C, green bars) that could limit virus infection. Subsequently and as a proof of concept, we evaluated the virus susceptibility of the cells using Reo-3 and EMCV. We found that the Trim24 and Gfi1 single knockout clones showed resistance to Reo-3 but moderate or no resistance against EMCV (Fig. 7A,B), compared to virus susceptible positive control cell lines (Supplementary Fig. S10). Therefore, we tested viability as a measure of cell death upon virus infection. However, the Gfi1 + Trim24 double knockout (Supplementary Fig. 7C) showed resistance to both viruses tested, even when cells were passaged and cultured for an additional week (Supplementary Fig. S11). Together these results show that eliminating repressors of the STAT1 regulatory network contributes to the antiviral potential of CHO cells. Further studies are currently being conducted to better understand the mechanisms by which the double KO cells are resistant to these RNA viruses, specifically focusing on the role of the identified innate immunity pathways in enhancing the survival of the host cells, such as (1) a potential deregulation of the type I IFN response upon sensing the virus (directly linked to the silencing of STAT1 transcription repressor genes), and (2) alternative inflammatory pathways related to the IL-1 family of cytokines (IL-33 and IL18) that may be regulated by the same repressor genes. This knowledge will be critical to ensure the safe use of virus-resistant engineer CHO cell lines in bioprocesses.

Our results suggest that the genomes of these RNA viruses are sensed by the same RIG-I/TLR3 receptors of the host cell, even if these RNA viruses of different families have found mechanisms to overcome the innate immune mechanisms of the CHO cells (Fig. 1). Activation of RIG-I/TLR3 with the ligand Poly I:C prior to virus infection gives an advantage to the host cell over the virus by inducing a robust type I IFN response allowing its survival. A similar outcome appears to be reached by deleting two of the type I IFN pathway negative regulators. The systems biology approach to identifying transcription factors impacting RNA virus infection could be replicated in the future for other virus classes, such as DNA viruses (e.g. MVM) which use other mechanisms for viral sensing such as TLR9, which is not expressed in CHO cells, therefore making CHO susceptible to MVM infection. Thus, using our approach, regulators of innate immunity could potentially be discovered to make DNA virus resistance cells by simulating TLR9 or its downstream activities in CHO cells with the use of CpG ODN to induce a TLR9-driven type I IFN response on the cell.

## Conclusions

Here we perform a genome-wide study of viral resistance in CHO, thereby demonstrating the utility of systems biology approaches to not only improve host cell productivity and metabolism^57–59^, but also to improve product safety. Specifically, we demonstrated that STAT1 and other key regulators are activated upon viral infection and/or poly I:C treatment, and that engineering the regulation of innate immunity aids in viral resistance. Studies have shown that modulating other genetic factors can promote viral resistance in CHO cells^10,60^. However, our findings suggest novel cell engineering targets beyond those coding for cell receptors. Thus, these insights provide further tools to enable the development of virus-resistant hosts to improve safety and secure the availability of biotherapeutic products^3,61,62^.

## Supplementary information

Additional file 1

Additional file 2

Additional file 3

Additional file 4

Additional file 5

Additional file 6

Additional file 7
