# Supplementary material for: Combating viral contaminants in CHO cells by engineering innate immunity
Source: Sci Rep. 2019 Jun 20;9:8827. doi: 10.1038/s41598-019-45126-x (PMC6586939; doi:10.1038/s41598-019-45126-x)
Supplement: Supplementary file 1 — Additional file 1 [file 41598_2019_45126_MOESM1_ESM.pdf]

Combating viral contaminants in CHO cells by engineering innate immunity

Austin W.T. Chiang, Shangzhong Li, Benjamin P. Kellman, Gouri Chattopadhyay, Yaqin Zhang, Chih-Chung Kuo, Jahir M. Gutierrez, Faezeh Ghazi, Hana Schmeisser, Patrice Ménard, Sara Petersen Bjørn, Bjørn G. Voldborg, Amy S. Rosenberg, Montserrat Puig and Nathan E. Lewis

Supplementary Information

Text

Text S1. Construction of plasmids to generate Gfi1, Trim24, and Gfi1+Trim24 knock-out cell lines. **04**

Text S2. Type I IFN protects CHO cells from VSV infection. **05**

Text S3. Gene set enrichment analysis (GSEA) and Upstream regulator (transcriptional factor) analysis. **07**

Text S4. Enrichment strength of type I interferon response. **09**

Text S5. Poly I:C pre-treatment of CHO cells protects against viral infection through the IFNβ-mediated pathway. **11**

Text S6. Identification of the STAT1 upstream regulators. **22**

Figures

Figure S1. IFNβ and Mx1 gene expression kinetics induced by poly I:C. **03**

Figure S2. Pre-treatment of the cell culture with type I IFN protein limits VSV infection. **06**

Figure S3. Enrichment strength of the interferon-alpha response. **10**

Figure S4. Poly I:C pre-treatment of CHO cells protects against viral infection through the IFNβ-mediated pathway. **12**

Figure S5. Differential induction of antiviral genes (Mx1 and IITMP3) by poly I:C and VSV or EMCV in contrast to Reo-3. …… **13**

Figure S6. Up-regulated DEGs present in m vs. Vp and m vs. p but not in m vs. Vm. …… **14**

Figure S7. NFATC2-dependent network in inducing STAT1 for inhibiting infection of mammalia. …… **16**

Figure S8. IRF3-dependent network in inducing STAT1 for inhibiting viral infection. …… **17**

Figure S9. Negative regulatory scores of STAT1 upstream regulators. …… **18**

Figure S10. Positive controls of susceptible CHO-S cell lines in the EMCV and Reo-3 virus infections. …… **19**

Figure S11. Long term culture of virus infection assay in CHO-S cell lines. …… **20**

Figure S12. Innate immunity genes in CHO cells are activated by poly I:C. …… **24**

Tables

Table S1. Statistics of differentially expressed genes. …… **21**

Table S2. The complete results of GSEA analysis in the comparison of m vs. Vm. …… **(EXCEL file)**

Table S3. The complete results of GSEA analysis in the comparison of m vs. p. …… **(EXCEL file)**

Table S4. The complete results of GSEA analysis in the comparison of Vm vs. Vp. …… **(EXCEL file)**

Table S5. A complete list of the identified upstream regulators in the comparisons of Vm vs. Vp. …… **(EXCEL file)**

Table S6. Enrichment results of the activated genes in the comparisons of m vs. p and m vs. Vp but not m vs. Vm for Reo-3. …… **(EXCEL file)**

Table S7. Enrichment results of the activated transcription factors in the comparison of Vm vs. Vp for Reo-3. …… **(EXCEL file)**

Table S8. Upstream regulators of STAT1 predicted by IPA. …… **23**

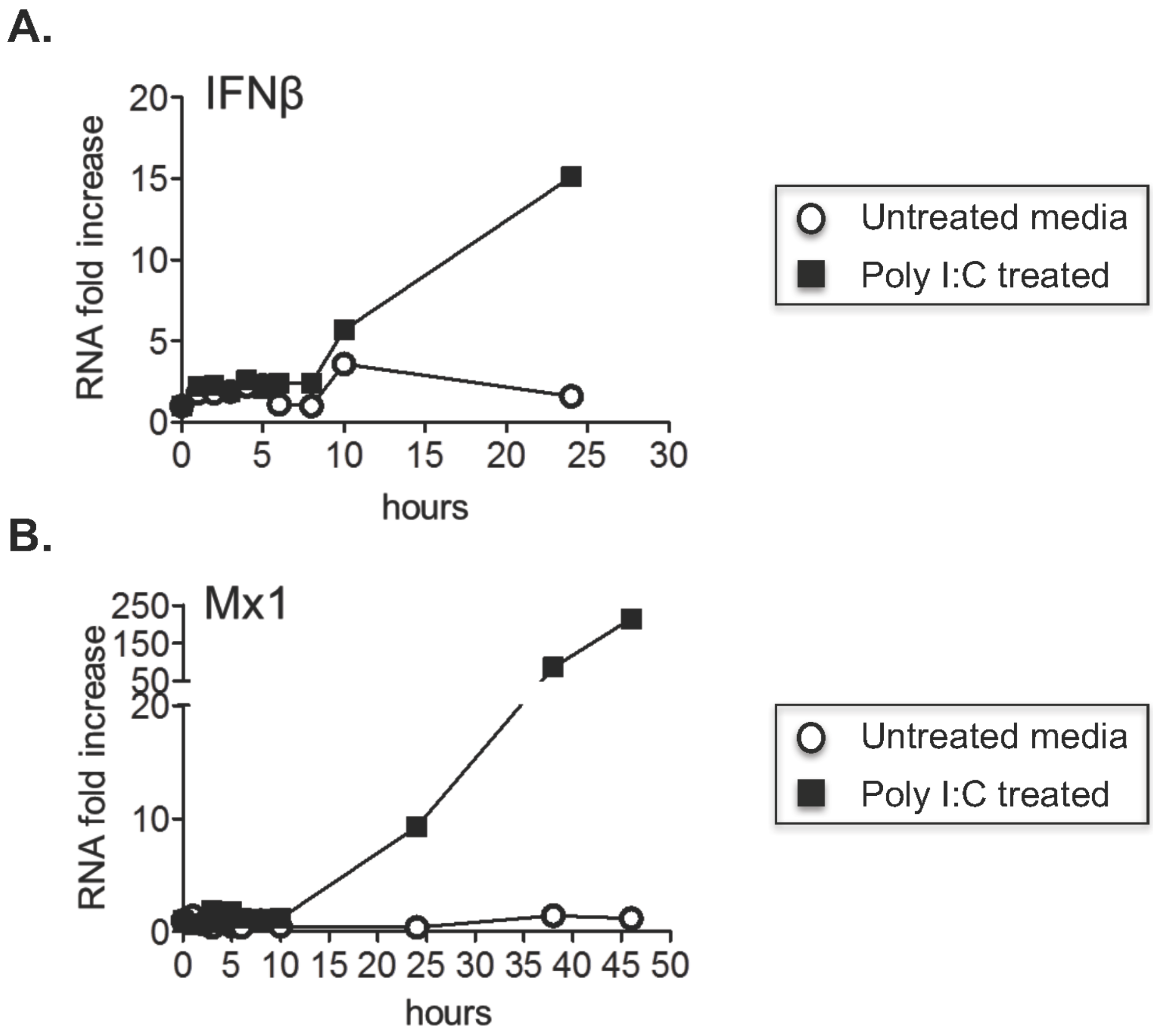

Figure S1. IFNβ and Mx1 gene expression kinetics induced by poly I:C. Changes in RNA transcript levels of anti-viral genes IFNβ (A) and Mx1 (B) in CHO cells treated with poly I:C (black squares) compared to untreated cultures (open circles) over time.

Text S1. Construction of plasmids to generate Gfi1, Trim24, and Gfi1+Trim24 knock-out cell lines

The plasmids we used to generate Gfi, Trim24, and Gfi+Trim24 knock-out cell lines are: Plasmids 2632 (GFP_2A_Cas9), Plasmids 6016 (Gfi1-665755) and 6018 (Trim24-1009774). The Plasmids 2632 (GFP_2A_Cas9) is described in (Grav et al., 2015). The Plasmids 6016 (Gfi1-665755) and 6018 (Trim24-1009774) were constructed as described in (Ronda et al., 2014) with the following modification: sgRNA plasmid sgRNA1_C described in (Ronda et al., 2014) was used as template in the PCR reaction to generate the backbone of gRNA plasmids.

Oligos used in the cloning reaction were:

17229	Gfi1-665755_gRNAfwd	GGAAAGGACGAAACACCGTGCGTGGAGCGGCCTCGCGGTTTTAGAGCTAGAAAT
17231	Trim24-1009774_gRNAfwd	GGAAAGGACGAAACACCGCACAAAAGACCACACCGTCGTTTTAGAGCTAGAAAT
17325	Gfi1-665755_gRNArev	CTAAAACCGCGAGGCCGCTCCACGCACGGTGTTTCGTCCTTTCCACAAGATAT
17327	Trim24-1009774_gRNArev	CTAAAACGACGGTGTGGTCTTTTGTGCGGTGTTTCGTCCTTTCCACAAGATAT

Primers used for MiSeq analysis were:

18484	Gfi1-1665755_MiSeqfwd	TCGTCGGCAGCGTCAGATGTGTATAAGAGACAGTGCACTGCCGGTAACTCTG
17423	Trim24-MiSeqfwd	TCGTCGGCAGCGTCAGATGTGTATAAGAGACAGGCAGTGCTAAAATACATCAGGGT
18485	Gfi1-1665755_MiSeqrev	GTCTCGTGGGCTCGGAGATGTGTATAAGAGACAGCTGCCCAGCACTCTAGAACC
17519	Trim24-11009774_MiSeqrev	GTCTCGTGGGCTCGGAGATGTGTATAAGAGACAGAGCTGTGAAGACAACGCAGA

Reference

Grav LM, Lee JS, Gerling S, Kallehauge T, H Hansen A, Kol S, Lee GM, Pedersen L, Kildegaard H. One-step generation of triple knockout CHO cell lines using CRISPR Cas9 and fluorescent enrichment. Biotechnol J. 2015; 10:1446–56.

Ronda, C., Pedersen, L. E., Hansen, H. G., Kallehauge, T. B. et al., Accelerating genome editing in CHO cells using CRISPR/Cas9 and CRISPy, a web-based target finding tool. *Biotechnol. Bioeng.* 2014, *111*, 1604–1616

Text S2. Type I IFN protects CHO cells from VSV infection

CHO cells failed to make a significant IFN response when infected with virus. It is well documented that type I IFN response is necessary to limit the extent of viral infection both in a cell culture and *in vivo*. Thus, we asked if the susceptibility to the virus was due to unresponsiveness of the cells to IFN rather than lack of ability to generate such a response. In order to simplify the screening, we first concentrated on VSV. Cells were seeded in 96-well plates and treated with human or murine type I IFN protein preparations at different dilutions (1:10) for 24h, prior to the addition of VSV (Figure S2). Infection progressed for 24h and cultures were stained with crystal violet (CV) to assess the extent of the protection by cytopathic effect. All IFN preparations limited viral cytopathic effect at the higher concentrations used (Figure S2). Of note, human IFNβ had the most potent anti-VSV effect of all the interferons tested, at least at the dose used in the experiment (Figure S2). These results indicate that CHO cells have a functional IFNα/β receptor and that its activation confers resistance of CHO cells to VSV infection.

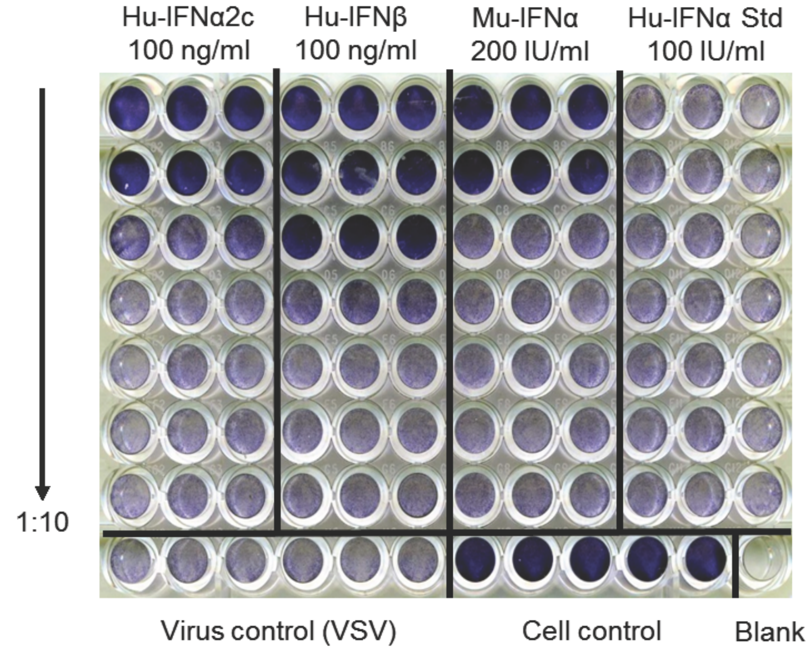

Figure S2. Pre-treatment of the cell culture with type I IFN protein limits VSV infection. Cells were cultured with the indicated concentration of human or murine IFN protein (serially diluted 1:10) for 24h prior to infection with VSV. Last row includes cells infected with VSV but not pretreated with IFN (Virus control) or non-infected cells (Cell control). The plate shows results from one experiment representative of 2, in which Hu-IFNα standard was used at 1000 IU/ml and gave comparable results.

Text S3. Gene set enrichment analysis (GSEA) and Upstream regulator (transcriptional factor) analysis

Gene set enrichment analysis (GSEA) and enrichment strength analysis

GSEA was performed using the Broad Institute GSEA software (Subramanian et al. 2005). A ranked list of genes (adjusted p-values < 0.05) was made using the differential expression values (Fold change in the log_2 scale) from differential gene expression analysis were run through the GSEA pre-ranked protocol. GSEA-pre-rank analysis was processed to detect significant molecular signature terms ('Hallmark' (50) and 'Reactome' (674) gene sets from the MSigDB were used here) for the differential expressed genes (see Tables S2-S4). Note that, the criteria for considering a molecular signature term as significant are: 1) after Benjamini-Hochberg false discovery correction, molecular signature terms with adjusted p-values less than 0.05; and 2) there are >30 genes presented in our gene list of this molecular signature terms.

The leading edge analysis allows for the GSEA to determine which subsets (referred to as the leading edge subset) of genes contributed the most to the enrichment signal of a given gene set's leading edge or core enrichment (Subramanian et al. 2005). The leading-edge analysis is determined from the enrichment score (ES), which is defined as the maximum deviation from zero. The enrichment strength describes the strength of the leading-edge subset of a gene set (i.e., the interferon-alpha response in this study) (Subramanian et al. 2005). Specifically, if the gene set is entirely within the first *N* positions in the ranked differentially expressed gene list, then the signal strength is maximal or 100%. If the gene set is spread throughout the list, then the signal strength decreases towards 0%.

Subramanian, A. et al. Gene set enrichment analysis: a knowledge-based approach for interpreting genome-wide expression profiles. *Proc Natl Acad Sci U. S. A.* **102**, 15545-15550 (2005).

Upstream regulator (transcriptional factor) analysis

The upstream regulators were predicted using the Ingenuity IPA Upstream Regulator Analysis Tool by calculating a regulation Z-score and an overlap p-value (Kramer et al. 2014), which were based on the number of known target genes of interest pathway/function, expression changes of these target genes and their agreement with literature findings. It was considered significantly activated (or inhibited) with an overlap p-value less than 0.05 and an IPA activation $|Z\text{-score}| \geq 1.96$. Note that, the criteria for generating the resulting table (Table 2) from IPA are: 1) Total nodes >= 10, and 2) Consistency score >= 5.00. Consistency score is an IPA measurement (Kramer et al. 2014) for measuring the consistency of a predicted network (capturing regulator-target-function relationships) from RNA-Seq data with literature knowledge. The higher consistency scores of the predicted regulatory networks denote better consistency with literature support than the predicted regulatory networks with lower consistency scores.

Kramer, A., Green, J., Pollard, J., Jr. & Tugendreich, S. Causal analysis approaches in Ingenuity Pathway Analysis. *Bioinformatics* **30**, 523-530 (2014).

Text S4. Enrichment strength of type I interferon response

Figure S3A shows that the enrichment strength (65%; see Text S2 for detailed explanation of enrichment strength) of ‘interferon alpha response’ from the comparison of untreated media and Reo-3 infected CHO cells (m vs. Vm) is smaller than those from the comparison of both virus presenting and poly I:C pretreated media (Vm vs. Vp; 77% and 77% for VSV and EMCV, respectively), which suggests that Reo-3-induced interferon alpha response might be insufficient for CHO cells limiting Reo-3 infection. Indeed, Reo-3 has been known to inhibit the type I IFN response using different strategies [40], such as modulation of cell RNA sensors (RIG-I and MDA5) and transcription factors (IRF3 and NF-kB) involved in induction of IFN. In consistence with our results, the IRF3 (z score = 4.96 and $p\text{-value} < 0.05$; Figure 1E) and NFkB pathways ($p\text{-value} = 1.12\times10^{-2}$ and NES = 2.22; Table S1) have been observed to be activated in the comparison of m vs. Vm. While the underling mechanism of how these RNA viruses evade the (innate) immune system is still unclear, these data substantiate the inability of CHO cells to elicit protective anti-viral mechanisms by not mounting an effective protective (type I IFN) response. However, these data suggest that viral infection could likely be limited by further inducing IFN pathways.

Figure S3B further demonstrates that temporal difference might be another factor accounting for the variations of type I interferon response. Indeed, we observed the enrichment strength of ‘interferon alpha response’ in the comparison of untreated cells and poly I:C pretreated cells (m vs. p) are different (73%, 70% and 78% for 30, 54 and 78 h, respectively). These differences might also result in the different magnitudes of downstream pathway/hallmark responses (Figure 2D) and upstream regulator expression variations (Figure 2E) across the different batches of samples that were collected from different time points.

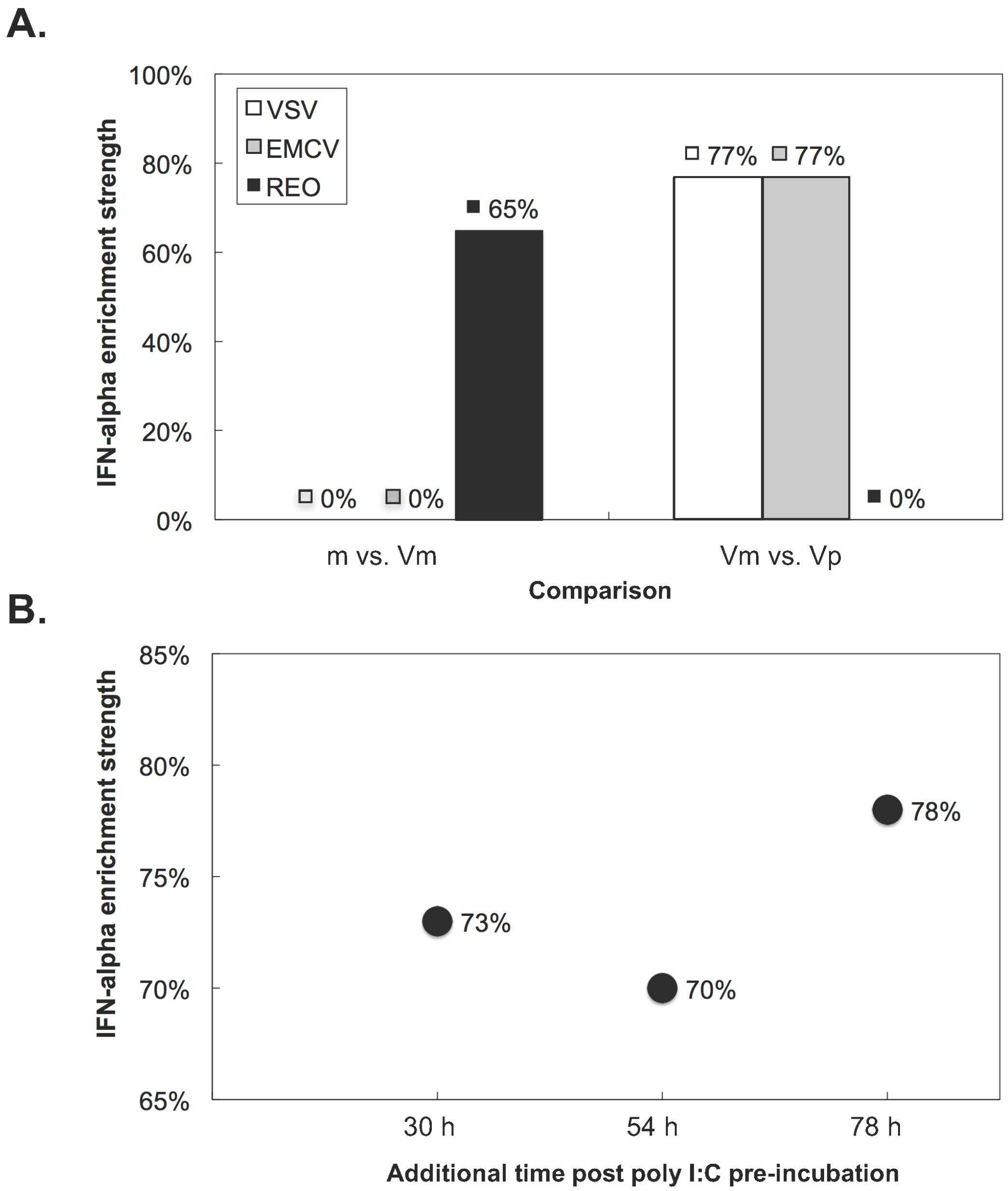

Figure S3. Enrichment strength of the interferon-alpha response. (A) Interferon-alpha response in the comparisons of m vs. Vm and Vm vs. Vp. (B) Time course of the effects of poly I:C on the Interferon-alpha response on non-infected cells pre-cultured with poly I:C for 16h. The 'interferon-alpha response' is a hallmark gene set of the gene set enrichment analysis (GSEA). The enrichment strength describes the leading-edge subset of a gene set (i.e., the interferon-alpha response in this study) [73]. If the gene set is entirely within the first *N* positions in the ranked differentially expressed gene list, then the signal strength is maximal or 100%. If the gene set is spread throughout the list, then the signal strength decreases towards 0%.

Text S5. Poly I:C pre-treatment of CHO cells protects against viral infection through the IFNβ-mediated pathway.

We next examined if the type I IFN response induced by poly I:C could protect CHO cells from RNA virus infections by evaluating effect of poly I:C on CHO susceptibility to VSV infection. Cells were cultured with 1μg/ml of poly I:C for 24h prior to infection with VSV (MOI of 0.1). As in previous experiments, the control poly I:C-treated CHO cell monolayer remained intact during the length of the experiment (48h) indicating that poly I:C per se was not toxic for the cells (Figure S4A). In contrast, disruption of the CHO cell monolayer was evident in wells where VSV was added, but not in wells where CHO cells were pre-incubated with poly I:C (Figures S4A and S4B). Moreover, the poly I:C-induced anti-viral response of the cell was IFNβ-dependent, as demonstrated by addition of a neutralizing antibody to IFN-β (Figure S4B). These results suggest that poly I:C treatment provides the cell with an advantageous immune state by activating the IFNβ-mediated pathway that counteracts viral escape mechanisms and results in cell survival.

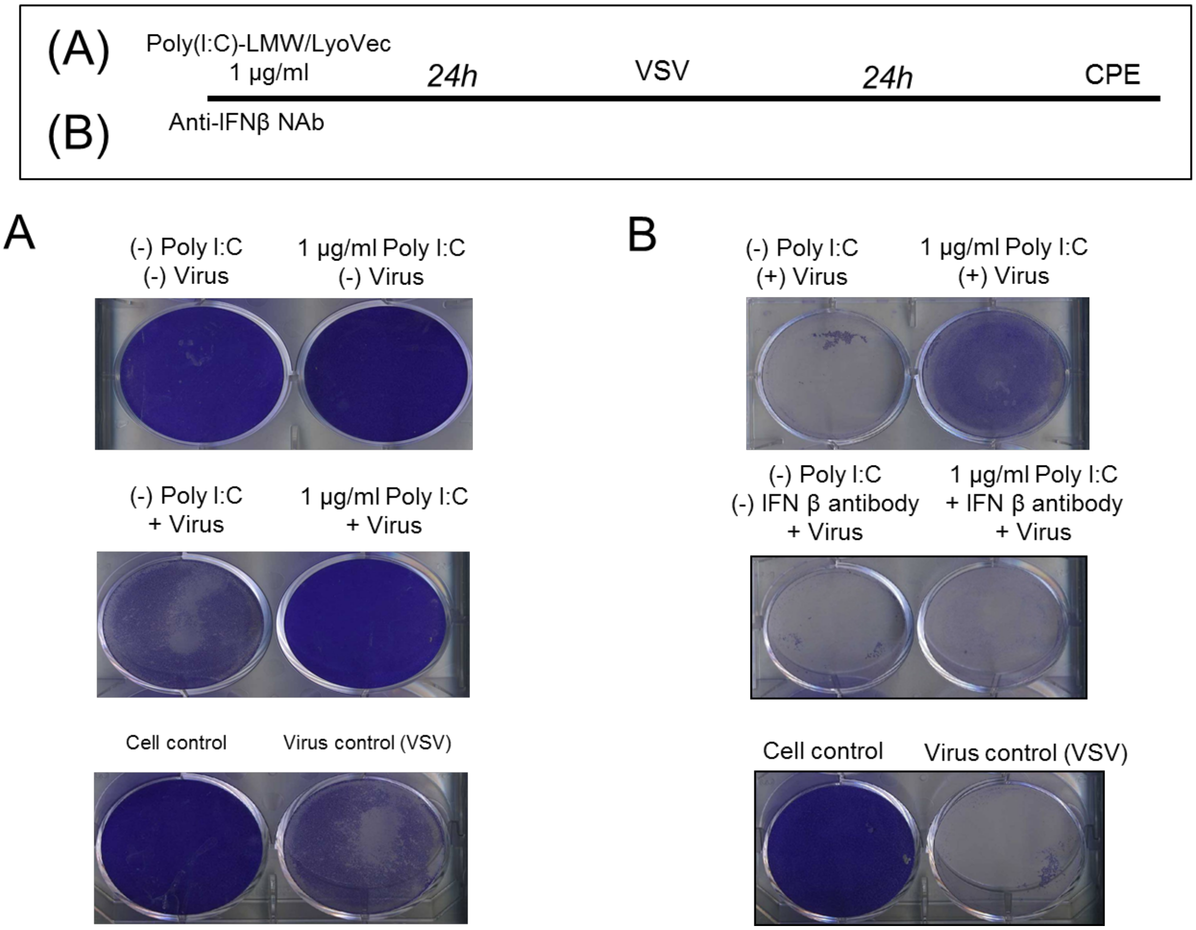

Figure S4. Poly I:C pre-treatment of CHO cells protects against viral infection through the IFNβ-mediated pathway. (A) Poly I:C induces effective anti-viral mechanisms in CHO cells. (B) IFNβ plays a protective role in the VSV infection, as treatment with anti-IFNβ neutralizing Ab prevents cell survival promoted by poly I:C.

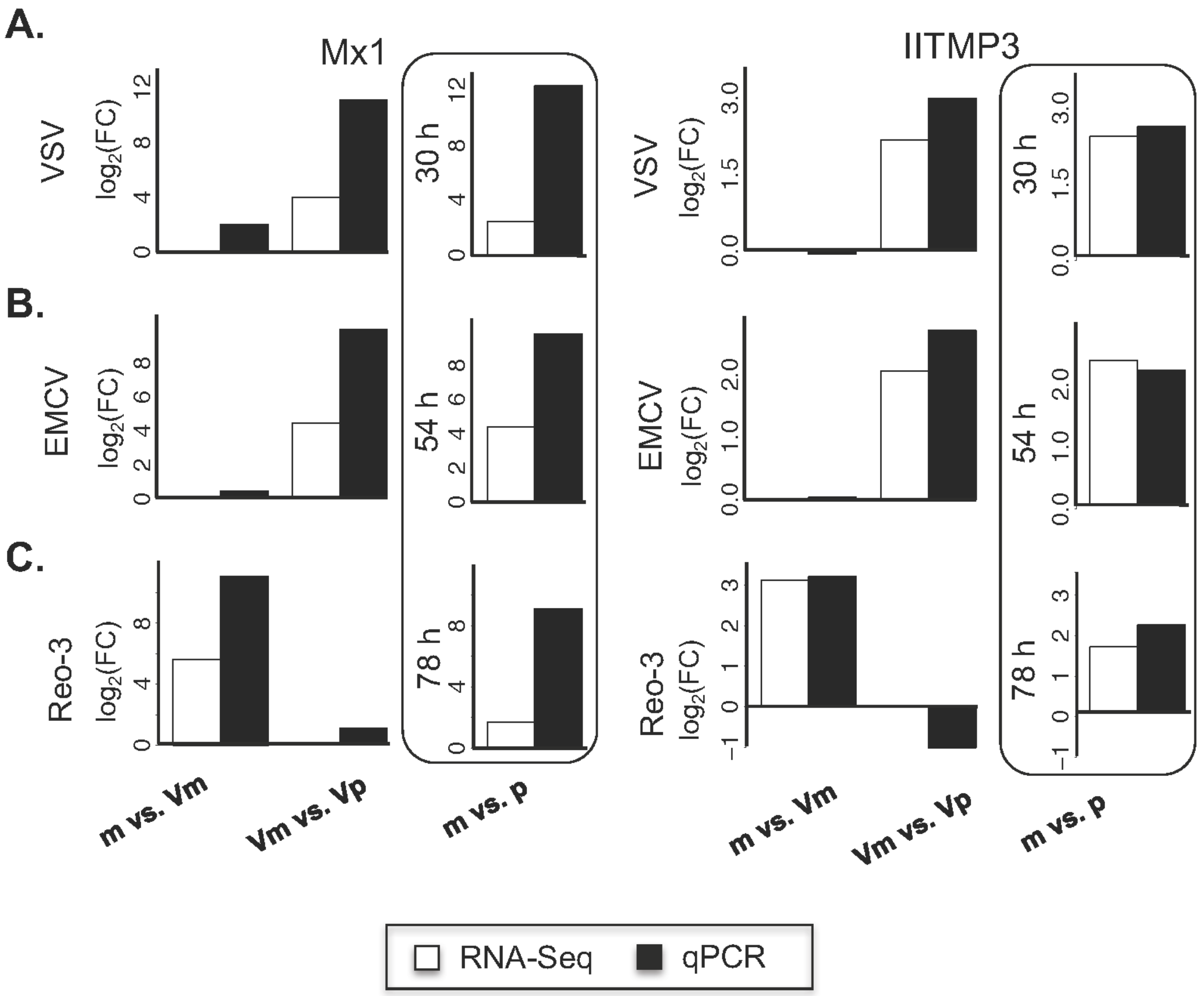

Figure S5. Differential induction of antiviral genes (Mx1 and IITMP3) by poly I:C and VSV or EMCV in contrast to Reo-3. Expression levels of Mx1 and IITMP3 were measured by Taqman real-time PCR (qPCR) and RNA-Seq. The x-axis represents the two culture conditions being compared. The y-axis denotes the $\log_2$ values of fold change ($\log_2$(FC)). RNA-Seq data was obtained using the R package of DESeq2.

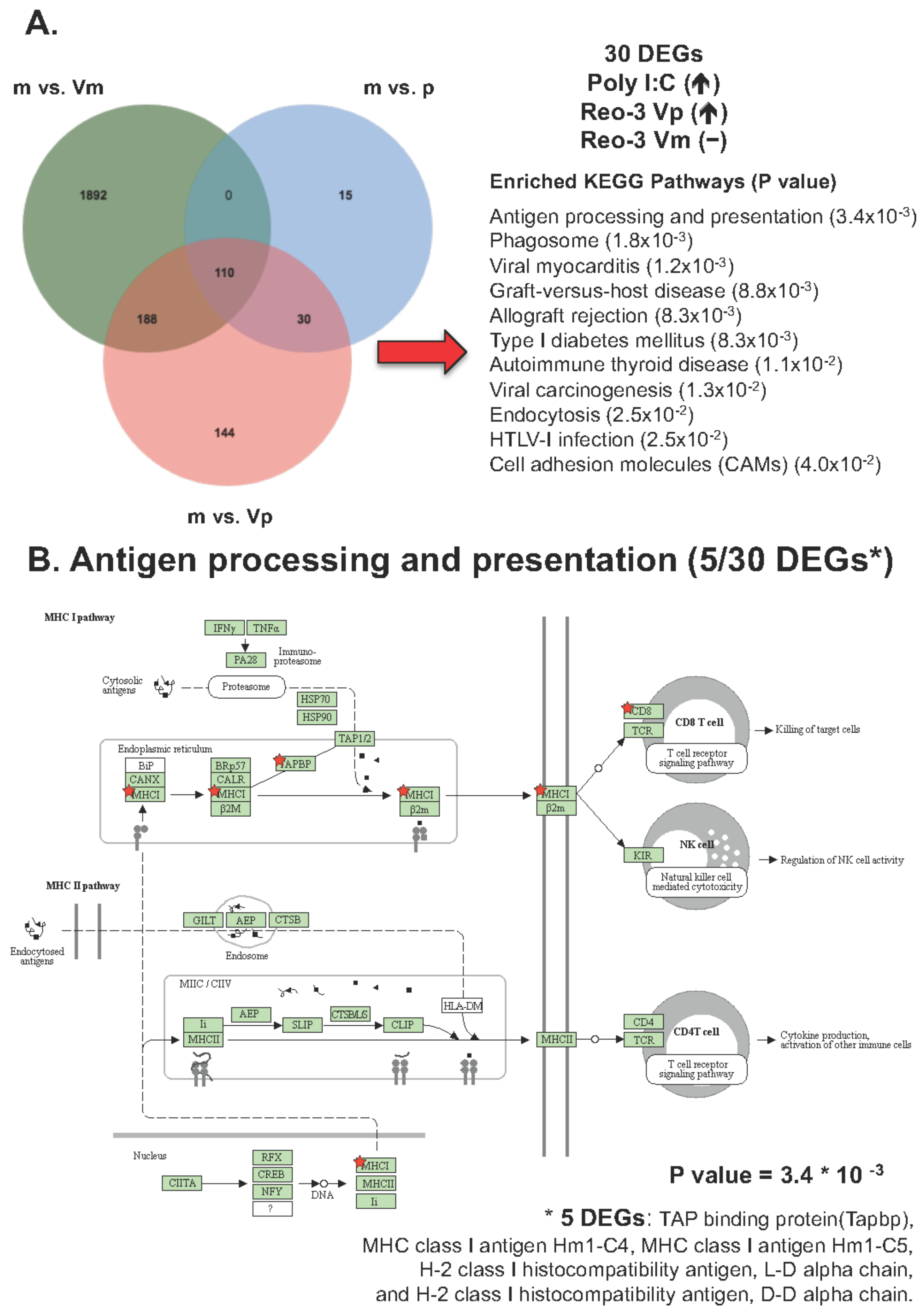

Figure S6. Up-regulated DEGs present in m vs. Vp and m vs. p but not in m vs. Vm. (A) Venn diagram of up regulated genes across different comparisons and the enriched KEGG pathways for the

30 DEGs that present with poly I:C treatment but not in Reo-3 infection. (B) Example of the most enriched KEGG pathway (Kanehisa et al. 2019): "antigen processing and presentation – homo sapiens (human) (hsa04612)" for the 30 DEGs. Note that, the criteria for identifying up regulated DEGs are: adjust p-value < 0.05 and fold change > 1.5 in the differential expressed genes test using DESeq2.

Kanehisa, M., Sato, Y., Furumichi, M., Morishima, K., and Tanabe, M.; New approach for understanding genome variations in KEGG. Nucleic Acids Res. 47, D590-D595 (2019).

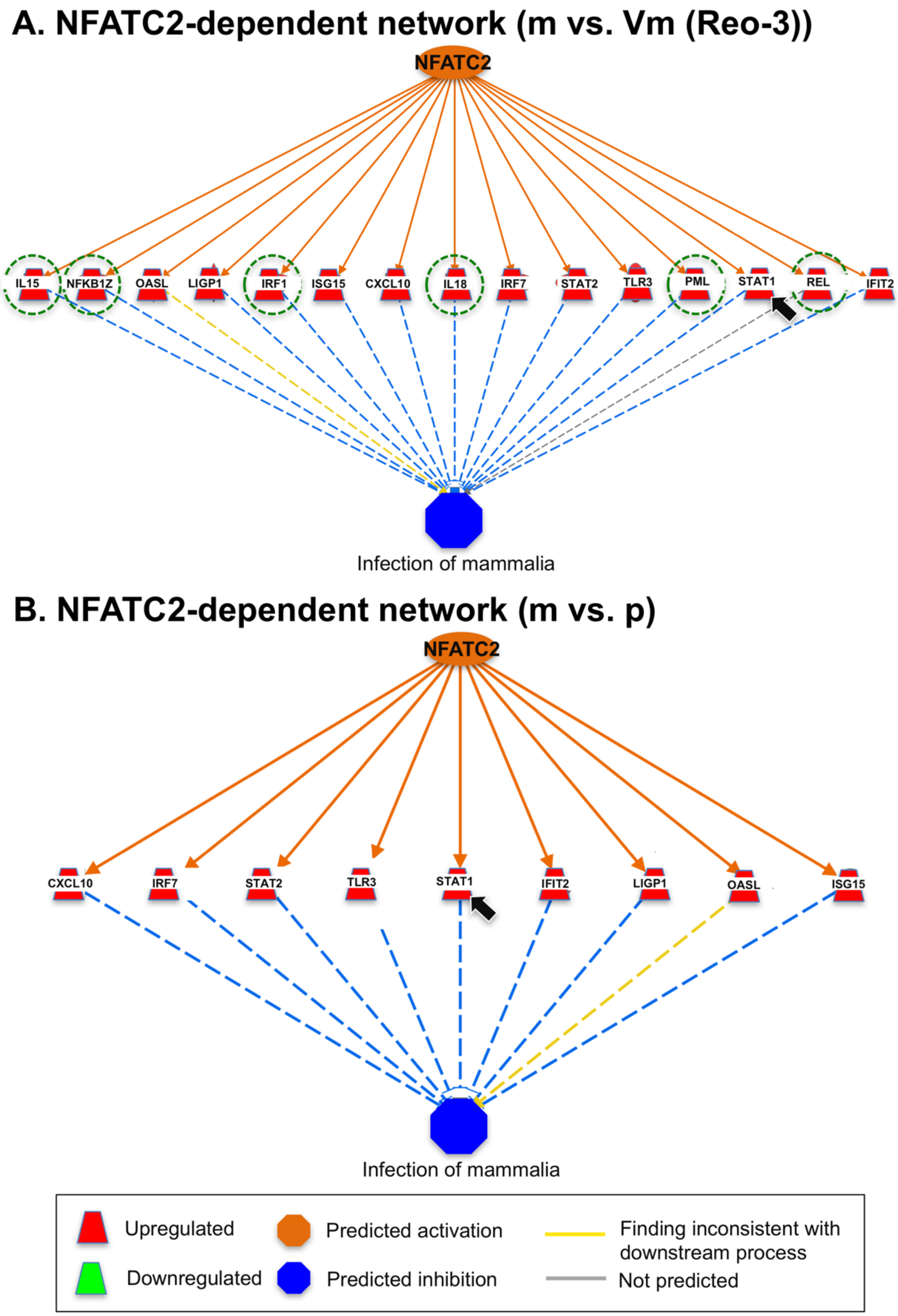

Figure S7. NFATC2-dependent network in inducing STAT1 for inhibiting infection of mammalia. (A) m vs. Vm. (B) m vs. p. Note that, the six genes (IL15, NFKB1Z, IRF1, IL18, PML and REL) that are different in these two networks are highlighted in the green dashed circles.

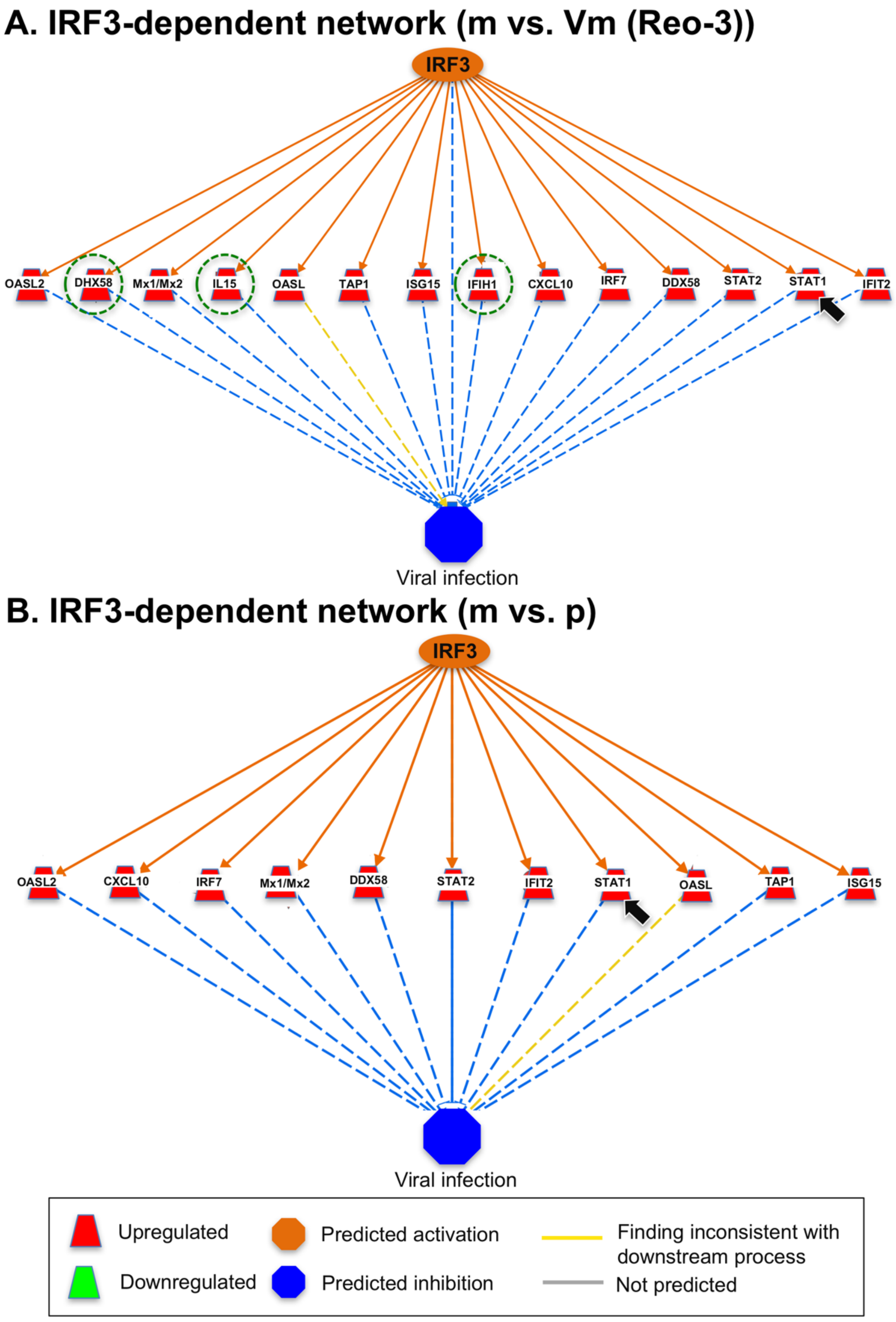

Figure S8. IRF3-dependent network inducing STAT1 for the inhibition of viral infection. (A) m vs. Vm. (B) m vs. p. Note that, the three genes (DHX58, IL15 and IFIH1) that are different in these two networks are highlighted in the green dashed circles.

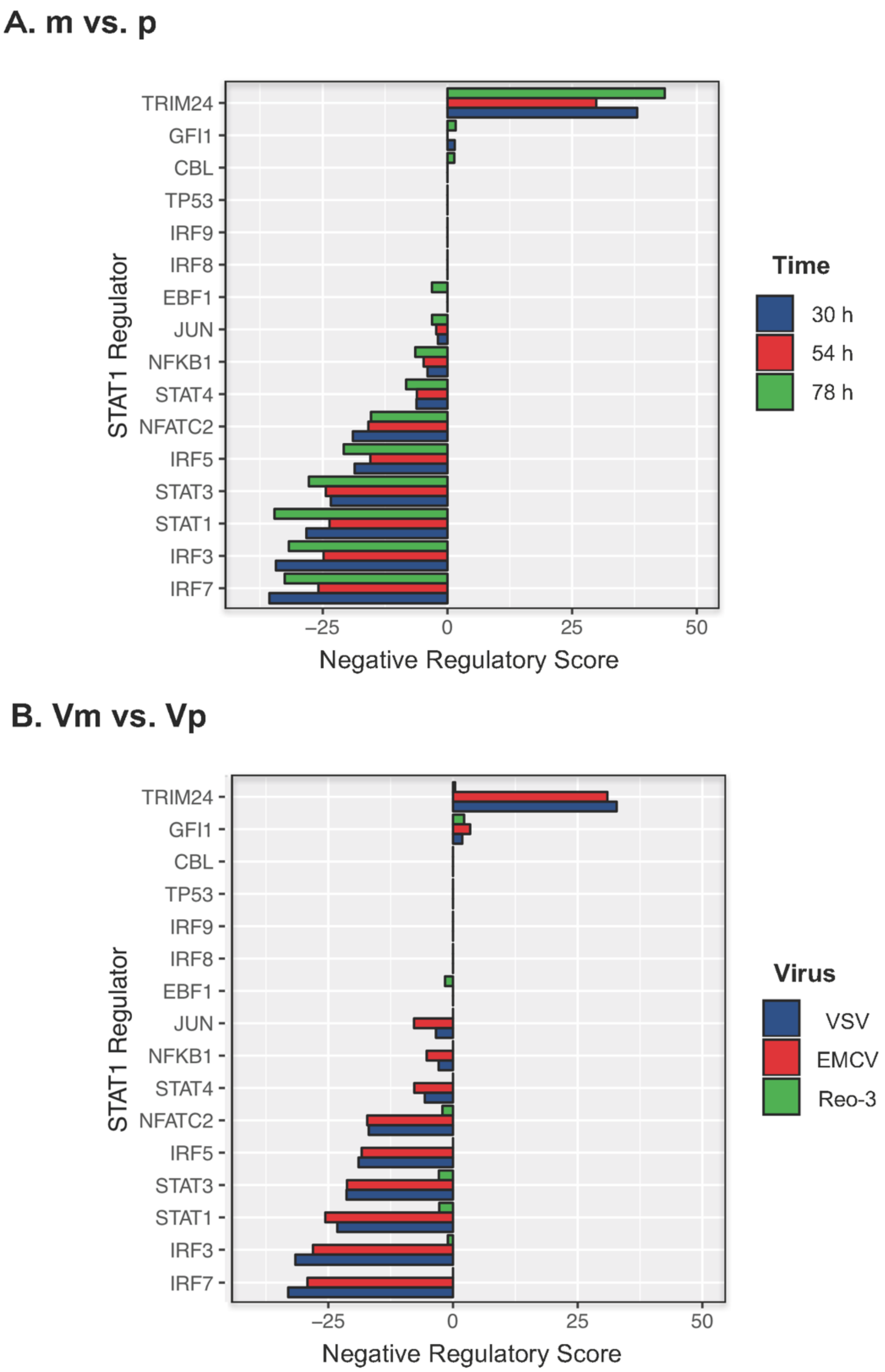

Figure S9. Negative regulatory scores of STAT1 upstream regulators. (A) Negative regulatory score (see details in Text S5) of STAT1 upstream regulators in the comparison of m (Media) vs. p (poly I:C). (B) Negative regulatory score of STAT1 upstream regulators in the comparison of Vm (Virus infected) vs. Vp (Virus infected in poly I:C treated media).

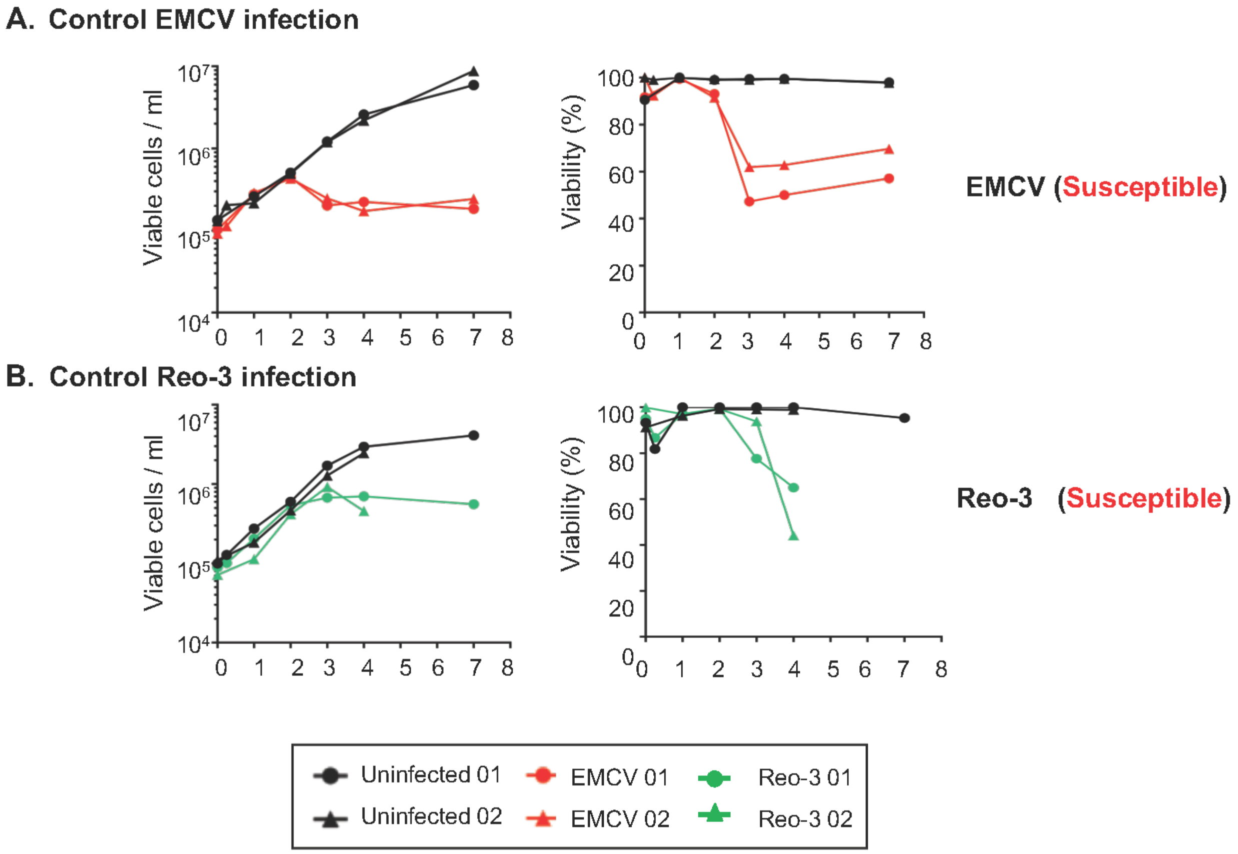

Figure S10. Positive controls of susceptible CHO-S cell lines in the EMCV and Reo-3 infections. Susceptible CHO cell lines were used as positive controls for EMCV (A) and Reo-3 (B) infections (see Figure 6) during the first seven days.

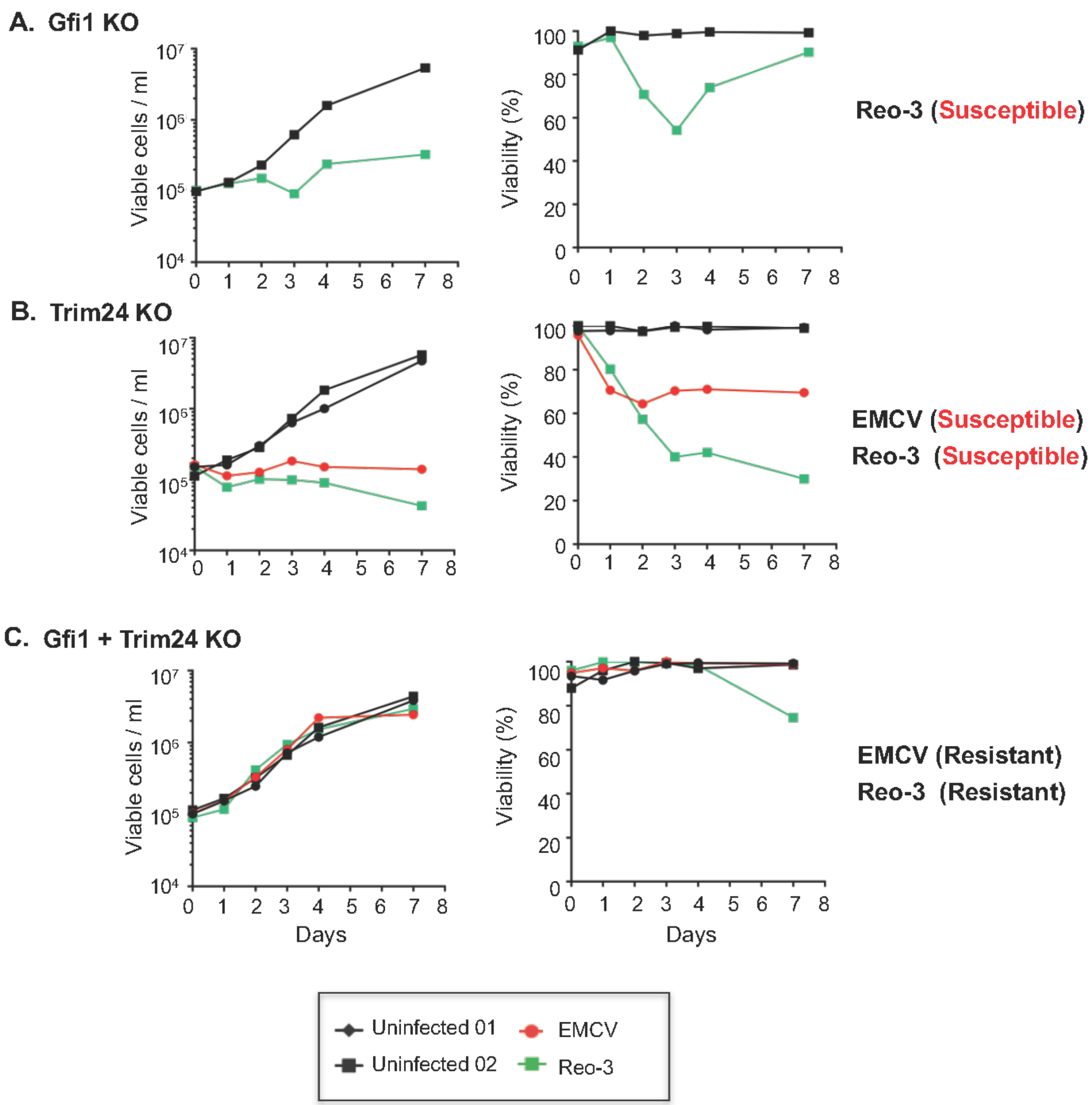

Figure S11. Extended culture of virus infected cells in CHO-S cell lines. Resistant cultures to EMCV (red circles) or Reo-3 (green squares) were passaged and followed up for an additional week for Gfi1 single knockout cells (A) and Trim24 single knockout cells (B), and Gfi1 and Trim24 double knockout cells (C). Note that Gfi1 KO cells infected with EMCV did not survive after the first week of culture. Uninfected cell density and viability are shown in black.

Table S1. Statistics of differentially expressed genes.

Comp.	Differential expression*	VSV		EMCV		Reo-3	
		Down	Up	Down	Up	Down	Up
1	m vs. Vm	1	24	8	16	1688	1945
2	Vm vs. Vp	271	281	275	337	1859	1657
		30 h		54 h		78 h	
		Down	Up	Down	Up	Down	Up
3	m vs. p	58	245	269	422	28	136

* m: untreated – uninfected (media control); p: poly I:C treated – uninfected; Vm: untreated – virus infected; Vp: poly I:C treated – virus infected. (Note that the criteria for identifying DEGs were: adjusted p-value < 0.05, and |Fold Change| > 1.5.)

Text S6. Identification of the STAT1 upstream regulators

We identify the upstream regulators of STAT1 as follows: First, using the IPA Upstream Regulator Analysis Tool, we obtained all the predicted upstream regulators in the RNA-Seq data comparisons: m vs. p and Vm vs. Vp. From those, we further identified upstream regulators with potential to regulate STAT1 gene using literature evidence (Table S8). Finally, we defined the negative regulatory score as shown below:

$$\text{Negative regulatory score} = -\text{log10}(\text{P} - \text{value}) \times Regulatoion\ Direction$$

$$Regulation\ Direction = \begin{cases} 1\ (Repressor) \\ 0\ (Unknown) \\ -1\ (Activator) \end{cases}$$

The p-value (Table S8) here is calculated using Fisher's Exact Test for measuring whether there is a statistically significant overlap between the differentially expressed genes in our dataset genes and the genes that are regulated by a TF, as reported in IPA. The higher negative regulatory score of a TF represents the larger potential in inhibiting STAT1 based on the RNA-Seq differential expression data (Figure S9).

Table S8. Upstream regulators of STAT1 predicted by IPA.

TF	m vs. p			Vm vs. Vp			Regulation Direction	Reference (PMID)
	30 h	54 h	78 h	VSV	EMCV	Reo-3		
TRIM24	8.9E-39	1.5E-30	2.6E-44	1.5E-33	1.1E-31	3.8E-01	Inhibited	21768647
IRF7	2.6E-36	1.6E-26	3.0E-33	9.0E-34	6.5E-30		Activate	23300459
IRF3	5.1E-35	1.4E-25	2.0E-32	2.5E-32	8.4E-29	9.4E-02	Activate	23300459
STAT1	6.5E-29	2.3E-24	2.6E-35	6.2E-24	2.5E-26	1.8E-03	Activate	22171011; 24412616
STAT3	4.1E-24	4.0E-25	2.1E-28	4.4E-22	5.5E-22	1.5E-03	Activate	12060750; 12060750
NFATC2	1.1E-19	1.4E-16	4.6E-16	1.3E-17	6.3E-18	7.5E-03	Activate	22078882
IRF5	2.4E-19	3.4E-16	1.6E-21	1.2E-19	4.9E-19		Activate	23300459
STAT4	6.2E-07	7.3E-07	5.2E-09	2.2E-06	1.7E-08		Activate	22968462
IRF9	1.2E-05	4.1E-04	2.8E-06	7.8E-05	2.5E-04		Unknown	20089923
IRF8	1.0E-04	1.4E-04	2.9E-06	2.4E-04	6.5E-06	3.7E-02	Unknown	22805310
NFKB1	1.0E-04	1.7E-05	3.5E-07	1.3E-03	5.3E-06		Activate	14568969
TP53	1.1E-02	7.9E-04	1.1E-03	7.8E-04	6.4E-05	7.2E-02	Unknown	16611991
JUN	1.2E-02	5.5E-03	8.0E-04	3.8E-04	1.5E-08		Activate	20436908
GFI1	3.5E-02		2.2E-02	1.4E-02	3.7E-04	6.0E-03	Inhibited	20547752
EBF1			7.7E-04			2.5E-02	Activate	24174531
CBL			4.3E-02				Inhibited	11704862

Note that, the number in each virus column denote p-value of the enrichment (hypergeometric) of the differentially expressed TF target genes in that TF.

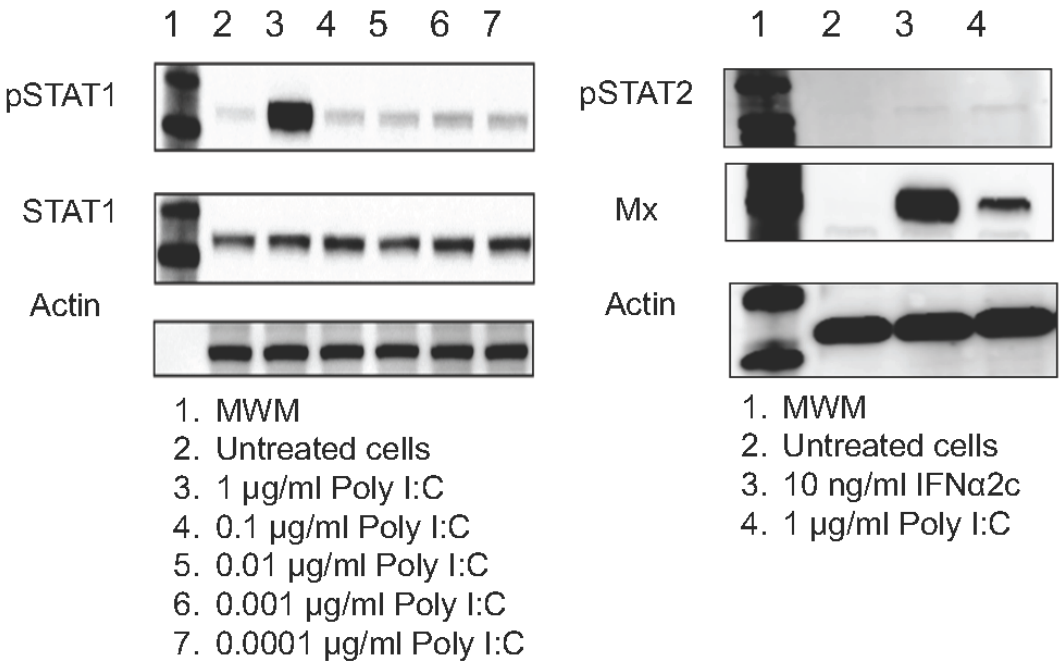

Figure S12. Innate immunity genes in CHO cells are activated by poly I:C. (Left panel) Poly I:C triggered STAT1 phosphorylation in a dose dependent manner, and (Right panel) the levels of Mx1 protein expression were comparable to those triggered by IFNα2c. Full images of western blot for main figures (Figure 2, (B) and (C)).
